# Early mannitol-triggered changes in the Arabidopsis leaf (phospho)proteome reveal growth regulators

**DOI:** 10.1093/jxb/ery261

**Published:** 2018-07-13

**Authors:** Natalia Nikonorova, Lisa Van den Broeck, Shanshuo Zhu, Brigitte van de Cotte, Marieke Dubois, Kris Gevaert, Dirk Inzé, Ive De Smet

**Affiliations:** 1 Ghent University, Department of Plant Biotechnology and Bioinformatics, Belgium; 2 VIB Center for Plant Systems Biology, Ghent, Belgium; 3 Ghent University, Department of Biochemistry, Ghent, Belgium; 4 VIB Center for Medical Biotechnology, Ghent, Belgium

**Keywords:** AHA2, *Arabidopsis thaliana*, CRRSP38, leaf growth, mild osmotic stress, phosphoproteome, signalling

## Abstract

Leaf growth is a complex, quantitative trait, controlled by a plethora of regulatory mechanisms. Diverse environmental stimuli inhibit leaf growth to cope with the perceived stress. In plant research, mannitol is often used to impose osmotic stress and study the underlying growth-repressing mechanisms. In growing leaf tissue of plants briefly exposed to mannitol-induced stress, a highly interconnected gene regulatory network is induced. However, early signalling and associated protein phosphorylation events that probably precede part of these transcriptional changes and that potentially act at the onset of mannitol-induced leaf size reduction are largely unknown. Here, we performed a proteome and phosphoproteome analysis on growing leaf tissue of *Arabidopsis thaliana* plants exposed to mild mannitol-induced stress and captured the fast (within the first half hour) events associated with this stress. Based on this in-depth data analysis, 167 and 172 differentially regulated proteins and phosphorylated sites were found. We provide these data sets as a community resource and we flag differentially phosphorylated proteins with described growth-regulatory functions, but we also illustrate potential novel regulators of shoot growth.

## Teaser

We captured early changes in the *Arabidopsis thaliana* growing leaf proteome and phosphoproteome upon mild mannitol stress and pinpointed novel regulators of shoot growth.

## Introduction

Plant growth is a very complex quantitative trait, involving multiple molecular players, crosstalk between hormones, and signalling pathways. As a plant continues to grow post-embryonically, it is exposed to a range of environmental conditions that can stimulate or adversely affect growth (Aguirrezabal *et al.*, 2006; Baerenfaller *et al.*, 2012; Plumb *et al.*, 2018). For example, plant growth and subsequently plant yield are drastically decreased as a result of drought (Boyer, 1982; Wang *et al.*, 2003). The regulation of a plant’s growth thus needs to be tightly controlled to sustain the post-embryonic development as well as the growth restrictions a plant might impose following stress exposure, such as drought. As a consequence, plant growth is under the control of a plethora of regulatory mechanisms.

In a temperate climate, water scarcity rarely threatens the survival of the plant, but rather reduces the growth and yield of the crop. Undoubtedly, the underlying molecular mechanisms differ depending on how severe the water limitation is, as plant lines that are more tolerant to severe stress rarely perform better under mild stress (Skirycz *et al.*, 2011*c*). Low concentrations of *in vitro* alternatives, such as mannitol, sorbitol, or polyethylene glycol (PEG), are used to study the molecular events leading to the growth inhibition associated with this stress condition (Verslues *et al.*, 2006). By transferring plants at a desired time point during development to an osmotic compound or by adding such a compound to liquid cultures, the very early signalling mechanisms underlying the stress-induced growth reduction can be revealed. Low concentrations of mannitol (25 mM) induce mild stress, triggering a decrease in *A. thaliana* rosette size of ~50% without affecting the plant’s development or survival (Claeys *et al.*, 2014). Mannitol can therefore be used as a growth-repressive compound and has been shown to be ideal for studying growth-regulating events (Skirycz *et al.*, 2011*c*; Van den Broeck *et al.*, 2017).

The osmotic stress responses in young growing leaves are very different from those in mature leaves. For example, in young shoots, mild stress induces the rapid accumulation of the ethylene precursor ACC (1-aminocyclopropane-1-carboxylate) and presumably also ethylene itself, instead of the classic drought-related hormone abscisic acid (ABA) (Skirycz *et al.*, 2011*a*). Unravelling the growth regulation upon stress is thus preferably studied in growing tissue instead of mature leaves or whole seedlings, as growing tissues are more subdued by growth-inhibitory mechanisms. In growing leaf tissue exposed to mannitol-induced stress, a highly interconnected gene regulatory network (GRN) is induced. The transcription factors that are part of this network regulate each other’s expression and have been shown to regulate leaf growth upon osmotic stress (Van den Broeck *et al.*, 2017). Some members of this GRN, such as ETHYLENE RESPONSE FACTOR 6 (ERF6), ERF9, and WRKY15, can activate the expression of *GIBBERELLIN2-OXIDASE6* (*GA2-OX6*), a gene encoding a gibberellin (GA) degradation enzyme (Rieu *et al.*, 2008). This presumably results in decreased levels of GA and DELLA protein stabilization, which pushes the cells permanently to exit the cell division phase and enter the cell differentiation phase (Claeys *et al.*, 2012). The first transcriptional changes occur very rapidly, after 40 min of stress. However, early signalling and associated phosphorylation events that precede part of these transcriptional changes are largely unknown, probably because most phosphoproteomic studies focused on severe, lethal stress or on whole seedlings, masking the growth-specific phosphorylation events (Bhaskara *et al.*, 2017*a*).

While the transcriptional events orchestrating leaf growth upon mild stress have been studied extensively, the early proteome and phosphoproteome changes are not yet fully understood. In this study, we performed proteome and phosphoproteome analyses on growing leaf tissue exposed to mannitol-induced stress and captured the rapid (within the first half hour) events associated with this stress. We demonstrate differences in proteome changes in the early (30 min) and later (4 h) mannitol-triggered proteome, such as the translational machinery and oxidation–reduction processes. Next, we evaluated the phoshoproteome and found several connections with the GA–DELLA pathway, which form interesting candidates for follow-up studies. We compared four phosphoproteome data sets after mild and severe stress, highlighting their distinct signalling pathways. Finally, genetic validation of some candidates for a growth phenotype revealed that CYSTEINE-RICH REPEAT SECRETORY PROTEIN 38 (CRRSP38) and H(+)-ATPASE 2 (AHA2) are potential novel regulators of leaf growth.

## Materials and methods

### Plant material and growth conditions

Wild-type plants (Col-0) were grown *in vitro* at 21 °C under a 16 h day (110 µmol m^−2^ s^−1^) and 8 h night regime. Sixty-four wild-type seeds were sown on a 14 cm diameter Petri dish with solid 1/2 Murashige and Skoog (MS) medium (Murashige and Skoog, 1962) (6.5 g l^−1^ agar, Sigma), overlaid with a nylon mesh (Prosep) of 20 μm pore size. During growth, plates were randomized. For the proteome (30 min and 4 h mannitol stress), phosphoproteome, and expression analyses, half of the plants were transferred to solid 1/2 MS medium (with 6.5 g l^-1^ agar) and the other half to solid 1/2 MS medium containing 25 mM mannitol (Sigma) at 15 days after stratification (DAS). The third leaf was harvested 20 min, 40 min, and 4 h (for expression analysis), 30 min (for proteomics and phosphoproteomics), or 4 h (for proteomics) after transfer. In total three or four biological replicates were performed for the 4 h proteomics and transcriptomics or the 30 min proteomics and phosphoproteomics, respectively. Approximately 100 mg of leaf material was harvested per sample. All experiments were performed independently.

### qPCR analyses

Samples were immediately frozen in liquid nitrogen and ground with a Retsch machine and 3 mm metal beads. Subsequently, RNA was extracted with TriZol (Invitrogen) and further purified with the RNeasy plant mini kit (Qiagen). For cDNA synthesis, the iScript cDNASynthesis Kit (Bio-Rad) was used with 1 µg of RNA as starting material. Quantitative real-time PCR (qRT-PCR) was performed with the LightCycler 480 Real-Time SYBR Green PCR System (Roche). The data were normalized against the average of housekeeping genes AT1G13320 and AT2G28390 (Czechowski *et al.*, 2005), as follows: dCt=Ct (gene)−Ct [average (housekeeping genes)] and ddCt=dCt (Control)−dCt (treatment). Ct represents the number of cycles at which the SYBR Green fluorescence reached a threshold during the exponential phase of amplification. Primers were designed with Primer-BLAST (https://www.ncbi.nlm.nih.gov/tools/primer-blast/) (Supplementary Tables S1, S2 at *JXB* online).

### Workflow of (phospho)proteomics

Plant material was flash-frozen in liquid nitrogen and ground into a fine powder. Subsequent proteome and phosphoproteome analyses were performed as previously described for protein extraction and trypsin digestion (Nikonorova *et al.*, 2018*a*) and phosphopeptide enrichment (Vu *et al.*, 2016) (see also Supplementary Protocol). For samples harvested 4 h after transfer to mannitol, protein extraction was performed by adding 100 µl of protein extraction buffer [5% ethylene glycol, 25 mM Tris–HCl pH 7.6, 15 mM MgCl_2_, 5 mM EGTA pH 8, 150 mM NaCl, 15 mM para-nitrophenylphosphate, 60 mM β-glycerophosphate, 1 mM DTT, 0.1% NP-40, 0.1 mM Na_3_VO_4_, 1 mM NaF, 1 mM phenylmethylsulphonyl fluoride (PMSF), 10 µg ml^−1^ leupeptin, 10 µg ml^−1^ aprotinin, 10 µg ml^−1^ soybean trypsin inhibitor, 0.1 mM benzamidine, 5 µg ml^−1^ antipain, 5 µg ml^−1^ pepstatin, 5 µg ml^−1^ chymostatin, 1 µM E64] and successive rounds of vortexing and freezing in liquid nitrogen. Cell debris was removed by centrifuging twice at 4 °C for 15 min at 20817 *g* and transferring the supernatans to a new tube. The sample preparation continued by a reduction/alkylation by adding 15 mM TCEP [tris(2-carboxyethyl)phosphine] and 30 mM iodoacetamide (IAA; freshly prepared), and the samples were incubated for 20 min at 37 °C in the dark. Afterwards the samples were desalted to get rid of the lysis buffer (containing protease inhibitors) and eluted in 666 µl of 50 mM ammonium bicarbonate. The protein concentration was then determined by a Bradford assay with 5 µl of the protein sample and 995 µl of 5× Bradford assay solution (200-fold dilution). Based on the calculated protein concentration, an adequate amount of protease (Endo-Lys C and trypsin mixture, Promega) was added in a 1:100 ratio and samples were incubated overnight at 37 °C. After digestion, the samples were completely dried down in a SpeedVac and re-suspended in a volume of loading solvent A (2% acetonitrile; 0.1% trifluoroacetic acid) to obtain a final concentration of 1 µg µl^−1^ peptide material. The samples were then centrifuged (16000 *g*, 4 °C, 15 min) to remove possible precipitate due to the acidic conditions. A 50 µl aliquot of the peptide mixtures was used for LC-MS/MS analysis. LC-MS/MS analysis was performed as previously described (Vu *et al.*, 2016). Proteome and phosphoproteome samples were analysed using 3 h gradients on a quadrupole Orbitrap instrument (Q Exactive).

MS/MS spectra were searched against the *A. thaliana* proteome database (TAIR10, containing 35386 entries; http://www.arabidopsis.org/) using the MaxQuant software (version 1.5.4.1). Settings for MaxQuant searches were set as previously described (Vu *et al.*, 2016). For the quantitative proteome and phosphoproteome analyses, the ‘ProteinGroups’ and ‘Phospho(STY)sites’ output files, respectively, generated by the MaxQuant search were loaded into Perseus software. For phosphoproteome data, only high-confidence hits with phosphorylation localization probability >0.75 were included in the analysis.

### Data filtering approach to identify relevant candidates for further studies

**Fig. 1.**
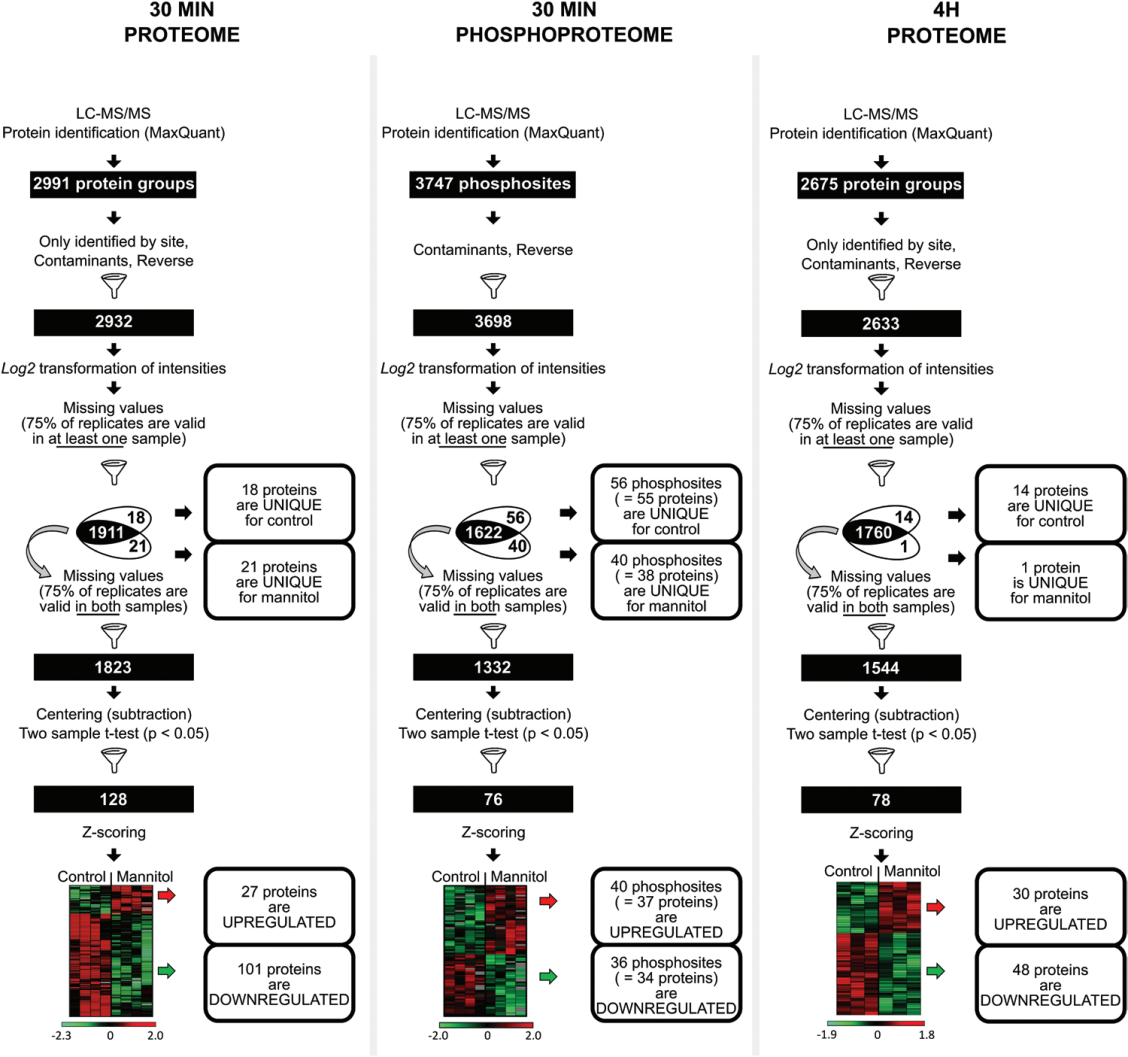
Mannitol-triggered changes in protein or phosphosite abundance upon 30 min and 4 h exposure. This workflow illustrates the steps to obtain a reliable set of proteins or phosphopeptides following LC-MS/MS. Venn diagrams indicate steps where unique proteins/phosphosites (with corresponding numbers) were filtered out from the statistical analysis. Heatmaps represent a hierarchical clustering of statistically significant proteins and phosphosites based on Pearson correlation. Centred *Z*-scored log2-transformed intensity values on heatmaps are colour-coded according to the colour gradient scales provided.

The original, complete data set containing log2-transformed intensities was split into three subsets, based on the percentage of replicates with missing or valid values per treatment with a threshold of 75% (Supplementary Fig. S1; Supplementary Protocol). The follow-up statistical workflow was performed as described previously (Vu *et al.*, 2016). In brief, the data set was centred around zero by a subtraction of the median within each replicate and subjected to a two-sample *t*-test (*P*<0.05) (Fig. 1).

### *In silico* analyses, data visualization, and statistics

Centred log2-transformed intensity values of the statistically significant proteins or phosphopeptides (or in some cases distinct phosphosites from the same phosphopeptide) were *Z*-scored, clustered into groups by a hierarchical clustering analysis based on Pearson correlation, and visualized as heat maps. To generate networks for known and predicted protein–protein interactions, the data sets were loaded to the STRING database (https://string-db.org; version 10.5) using a high confidence interaction score (>0.7). As active interaction sources, text mining, experiments, databases, co-expression, neighbourhood, gene fusion, and co-occurrence were selected. (Phospho)proteins that did not have any interaction were removed from the network. Biological Process terms were retrieved from the TAIR portal (www.arabidopsis.org, Bulk Data Retrieval). Further data visualization was performed in Cytoscape (version 3.5.1) on the extracted interaction network and Gene Ontology (GO) annotations. GO enrichment analysis was performed in the PLAZA 4.0 workbench using entire species as background model and 0.05 as a *P*-value cut-off. The HMMER web server was used for the prediction of kinase domains. For Motif-X analyses (Chou and Schwartz, 2011), the sequences (limited to 13 amino acids) of up- and down-regulated phosphopeptides were pre-aligned with the identified phosphosite centred. The IPI Arabidopsis Proteome was used as the background database. The occurrence threshold was set at the minimum of five peptides, and the *P*-value threshold was set at <10^−6^. Venn diagrams were created using the Venny 2.1 online tool (http://bioinfogp.cnb.csic.es/tools/venny). Barcharts, boxplots, and statistical analyses for qPCR and phenotyping data were performed using R software (https://www.R-project.org) and all figures were generated with Inkscape or Photoshop.

### Genotyping

The *crrsp38-1* (SALK_151902) and *aha2-4* (SALK_082786) mutant *A. thaliana* plants were obtained from Nottingham Arabidopsis Stock Centre (NASC). The homozygous *A. thaliana* mutants were identified by PCR using primers specific to the insertion T-DNA (Supplementary Table S3) and the LB primer (ATTTTGCCGATTTCGGAAC). The SALK lines used were in the Col-0 background.

### Leaf growth phenotyping

Both wild-type (Col-0) and mutant plants were grown *in vitro* on 14 cm diameter Petri dishes at 21 °C under a 16 h day (110 µmol m^−2^ s^−1^) and 8 h night regime. The mutant line was grown together with the appropriate control on one plate to correct for plate effects. For each condition (MS or mannitol), 4–6 plates with eight wild-type and eight mutant seeds per plate were sown. Half of the plants were grown on solid (9 g l^−1^ agar, Sigma) 1/2 MS control medium and the other half on solid 1/2 MS medium with the addition of 25 mM d-mannitol (Sigma). The plates were photographed at 16 or 22 DAS, and all images were analysed using ImageJ (Schindelin *et al.*, 2015) to measure the projected rosette area. In total, four independent experiments were performed for *aha2-4* and *crrsp38-1* at 22 DAS and two independent experiments for *aha2-4* at 16 DAS. To determine statistical significance of the raw data, an ANOVA test followed by a Tukey’s post-hoc test was performed (Supplementary Tables S4–S9).

## Results

### Proteome and phosphoproteome profiling to unravel the early mannitol response

To gain insight into the early molecular changes associated with mannitol-triggered growth inhibition, we focused on changes in the proteome and phosphoproteome in expanding leaf tissue of *A. thaliana*. We opted for low concentrations of the osmoticum mannitol (25 mM), which enabled a mild stress that does not affect plant survival but solely represses growth (Skirycz *et al.*, 2011*a*; Claeys *et al.*, 2014). Specifically, *A. thaliana* seedlings at 15 DAS were transferred to solid 1/2 MS medium containing 25 mM mannitol and, after 30 min, the third expanding leaf was harvested in four biological repeats. We chose the 30 min time point because this time point coincides with the earliest changes observed in the transcriptome data on mild osmotic stress in growing leaves (Van den Broeck *et al.*, 2017). Next, proteins were extracted and used for two parallel analyses: (i) the total proteome, enabling us to identify key proteins responding to mannitol; and (ii) the phosphoproteome, allowing us to gain insights into the early phosphorylation events. The proteome analysis of control and mannitol-treated samples resulted in the identification of 2932 protein groups in total (a protein group includes proteins that cannot be unambiguously identified by unique peptides but have only shared peptides) (Fig. 1; Supplementary Table S10). The phosphoproteome analysis led to the identification of 3698 phosphorylated peptides that could be mapped on 1466 proteins (Fig. 1; Supplementary Table S11). To address whether the early mannitol-triggered leaf proteome is significantly different from a more long-term exposure, we also performed a 4 h mannitol treatment. This time point was chosen as it is 2 h later than the maximum transcriptional induction of several key transcription factors that were previously identified under mild osmotic stress and at that time point downstream protein changes have most probably occurred (Van den Broeck *et al.*, 2017). The third expanding leaf was harvested in three biological replicates and led to identification of 2633 protein groups (Fig. 1; Supplementary Table S12).

To overcome the problem of missing values without imputation or complex statistical analysis (Koopmans *et al.*, 2014; Lazar *et al.*, 2016; Wang *et al.*, 2017), we applied a hybrid approach for data analysis that treats intensity-based and presence/absence data separately (Fig. 1; Supplementary Fig. S1; see also the Materials and methods, and Supplementary Protocol). Dividing the data set into three subsets allowed us to minimize the number of missing values in the input for regression analysis (subset 1), eliminate unreliable detections or quantifications (subset 2), and include unique proteins or phosphopeptides that are present in a treated sample and absent in the control conditions, or vice versa (subset 3) (Fig. 1; Supplementary Fig. S1; Supplementary Tables S10–12). To simplify data characterization further, we arranged proteins or phosphopeptides in two groups: up-regulated (detected only in mannitol-treated samples or significantly more abundant upon mannitol treatment) and down-regulated (detected only in control samples or significantly more abundant in control conditions) (from here on referred to as differentially regulated proteins or phosphopeptides).

### Early (30 min) mannitol-triggered effects on the leaf proteome

For the proteome analysis, the above-described approach resulted in 18 and 21 unique proteins detected only in the control and mannitol-treated sample, respectively (Fig. 1; Supplementary Table S10). In addition, statistical analysis determined 128 differentially abundant proteins: 27 that were found at higher abundance and 101 that were found at lower abundance upon mannitol treatment compared with the control (Fig. 1; Supplementary Table S10).

**Fig. 2.**
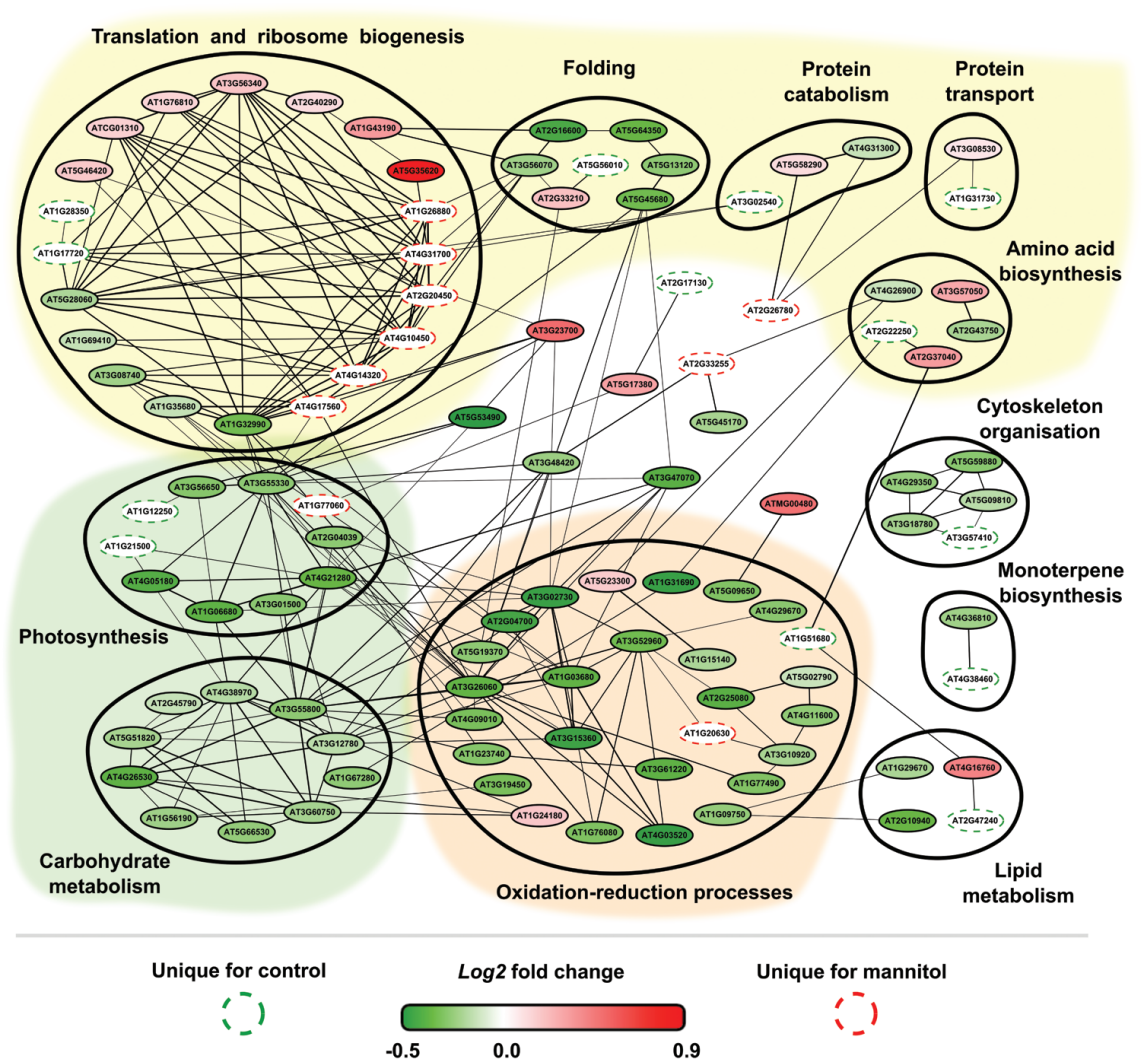
Functional protein association network of significant mannitol-regulated proteins (30 min treatment). GO annotations for biological process of differentially regulated proteins were superimposed on the network and nodes were grouped accordingly. Coloured backgrounds indicate functions related to protein metabolism (yellow), photosynthesis and carbohydrate metabolism (green), and oxidation–reduction processes (orange). Unique proteins were indicated with dashed lines, while differentially abundant proteins were coloured ranging from dark green to red depending on the log2 fold change. The thickness of connecting lines indicates a combined score of interaction.

Using the PLAZA 4.0 platform, GO enrichment analysis was conducted on the data set in the context of biological process (Supplementary Table S13). GO enrichment on biological processes showed that differentially regulated proteins (including unique proteins) were involved in various processes, such as cellular response to oxidative stress, response to cold and salt stress, hexose metabolic process, response to stress and abiotic stimulus, and response to cytokinin. To better understand the relationships between the 167 differentially regulated proteins, we constructed a functional protein association network consisting of 105 interacting proteins with 242 (potential) interactions (see the Materials and methods for details). To gain insight into the early biological processes affected by mild osmotic stress, GO annotations of differentially regulated proteins were superimposed on the network and nodes were grouped accordingly (Fig. 2). This approach revealed that most interacting proteins were involved in protein metabolism, specifically in amino acid biosynthesis, translation and ribosome biogenesis, protein folding, intracellular protein transport, and ubiquitin-dependent protein degradation.

In a first large group of differentially regulated interacting proteins, we observed that ribosomal proteins were highly regulated upon mannitol exposure, suggesting an altered capacity for protein translation. A second large group of proteins in our network was involved in oxidation–reduction processes and biotic and abiotic stress responses. CATALASE1 (CAT1) was detected as a unique protein for the mannitol-treated samples, while other detected reduction–oxidation proteins appeared to be down-regulated. The third largest cluster in the network consisted of proteins related to photosynthesis and carbohydrate metabolism. Members of this group were mainly down-regulated under mannitol stress, except for a PHOSPHOENOLPYRUVATE CARBOXYLASE family protein (PEPC, AT1G77060).

To conclude, our results point towards a down-regulation of photosynthesis and up-regulation of CAT1 probably for reactive oxygen species (ROS) scavenging; both systems potentially reduce ROS production. In addition, our results suggest an unexpected role for peroxidases in the early mannitol response as these were down-regulated.

### Effects of 4 h mannitol treatment on the leaf proteome

To get an idea of the changes in protein abundance upon more prolonged mild osmotic stress, we also performed a proteome analysis on growing leaves exposed to 4 h of mannitol treatment. The above-described data analysis workflow led to the identification of 15 unique proteins (based on absence or presence in all three biological replicates) and 78 significantly differentially abundant proteins (Fig. 1; Supplementary Table S12). GO enrichment on biological processes showed that differentially regulated proteins were, among several others, involved in stomatal movement, translation, photosynthesis, and response to abiotic stimulus (Supplementary Table S13). A functional protein association network was built for differentially regulated proteins (see the Materials and methods for details) and, similar to 30 min mannitol treatment, the vast majority of interacting proteins were involved in translation and ribosome biogenesis (Supplementary Fig. S2).

**Fig. 3.**
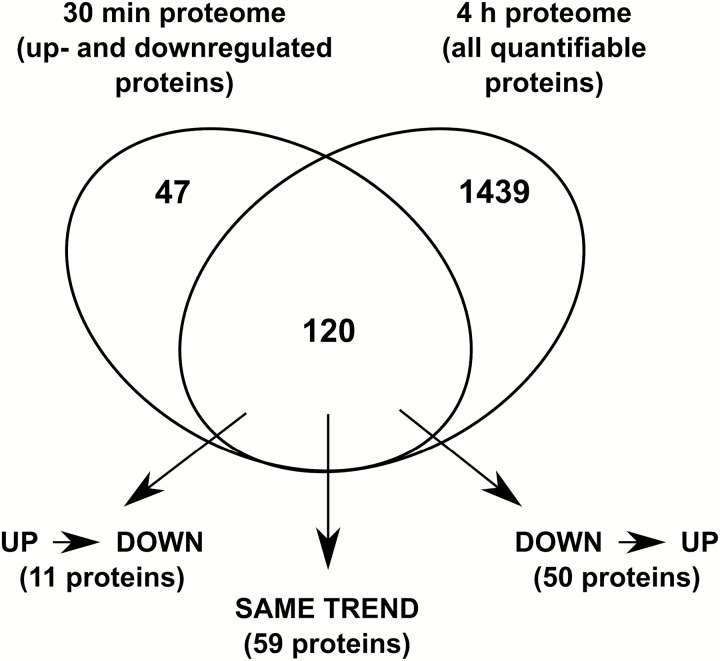
Venn diagram showing the overlap between the significant up- and down-regulated proteins from the 30 min proteome data set and all quantifiable proteins from the 4 h proteome data set. In the overlap, three subsets of proteins are identified based on the changes in their abundances from 30 min to 4 h of mannitol stress; ‘SAME TREND’ proteins are up- or down-regulated at both 30 min and 4 h, ‘UP→DOWN’ indicate proteins that are up-regulated after 30 min but down-regulated at 4 h, and ‘DOWN→UP’ indicates the opposite.

To unravel dynamic changes of proteins that were differentially regulated at 30 min, we tracked these proteins in the 4 h mannitol data set and compared protein fold changes. Thus, 167 differentially regulated proteins of the 30 min data set were mapped on the total 4 h proteome data set and 120 proteins were retained (Fig. 3; Supplementary Table S14). Of the 120 mapped proteins, 59 proteins showed a similar trend at 30 min and 4 h after mannitol treatment, and the remaining 61 proteins displayed a different trend. Overall, these observations suggested a dynamic control of protein levels during mild osmotic stress response.

### Lack of correlation between early mannitol-triggered transcript and protein fold changes

**Fig. 4.**
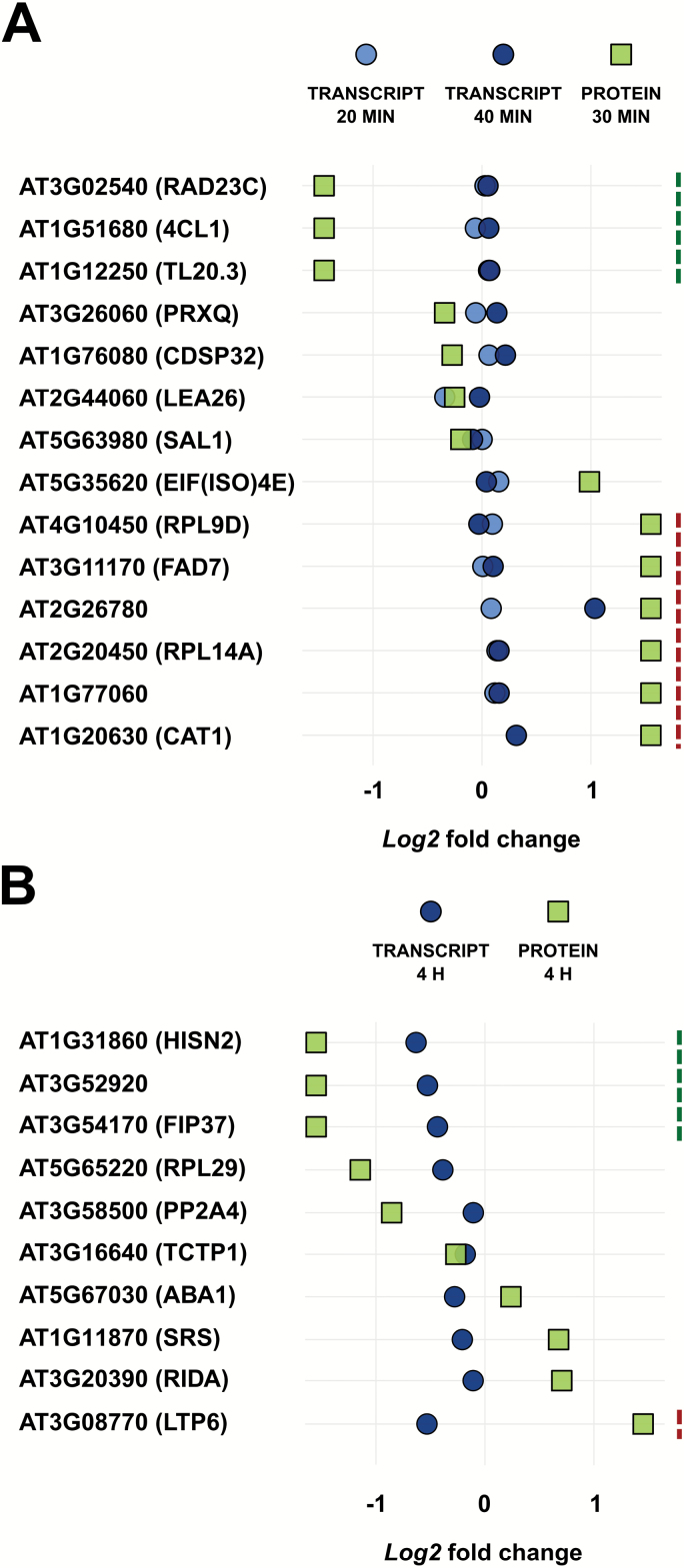
Comparison of protein abundance with the transcript level of the corresponding genes. The differential expression of genes encoding significant differentially up- or down-regulated proteins at (A) 30 min or (B) 4 h after mannitol treatment was analysed at (A) 20 min or 40 min and (B) 4 h after mannitol treatment. The expression and protein levels were measured in expanding leaf tissue upon mannitol treatment and compared with control conditions. Dashed lines indicate proteins unique for control (green) or mannitol-treated (red) samples. RAD23C, RADIATION SENSITIVE23C; 4CL1, 4-COUMARATE:COA LIGASE 1; TL20.3, THYLAKOID LUMENAL PROTEIN TL20.3; PRXQ, PEROXIREDOXIN Q; CDSP32, CHLOROPLASTIC DROUGHT-INDUCED STRESS PROTEIN OF 32 kDa; LEA26, LATE EMBRYOGENESIS ABUNDANT 26; SAL1, SAL1 phosphatase; EIF(ISO)4E, EUKARYOTIC TRANSLATION INITIATION FACTOR ISOFORM 4E; RPL9D, 60S RIBOSOMAL PROTEIN L9-2; FAD7, FATTY ACID DESATURASE 7; RPL14A, 60S RIBOSOMAL PROTEIN L14-1; CAT1, CATALASE 1; HISN2, HISTIDINE BIOSYNTHESIS 2; FIP37, FKBP12-INTERACTING PROTEIN OF 37 kDa; RPL29, 50S RIBOSOMAL PROTEIN L29; PP2A4, PROTEIN PHOSPHATASE 2A ISOFORM 4; TCTP1, TRANSLATIONALLY-CONTROLLED TUMOR PROTEIN 1; ABA1, ABA DEFICIENT 1; SRS, SERYL-tRNA SYNTHETASE; RIDA, REACTIVE INTERMEDIATE DEAMINASE A; LTP6, NON-SPECIFIC LIPID-TRANSFER PROTEIN 6.

Because some proteins are mainly regulated at the post-transcriptional level, we evaluated to what extent changes in transcript abundance for 14 genes from the 30 min proteome data set correlated with protein levels. We included the genes with the highest change in protein abundance and some of the unique proteins. Expanding leaf tissue was harvested 20 min and 40 min after mild mannitol treatment. Surprisingly, we found almost no obvious changes after 20 min and 40 min of mannitol treatment at the transcriptional level for the selected genes (Fig. 4A). This suggested that changes in protein levels at 30 min were not caused by changes in transcript abundance, but were more probably the result of differences in protein translation, degradation, or stabilization. Additionally, we evaluated the expression of 10 genes whose encoded proteins were differentially abundant after 4 h of mannitol treatment. As for the short-term expression analysis, no significant up- or down-regulation of the selected genes could be observed in growing leaves after 4 h of mild mannitol-induced stress (Fig. 4B). We concluded that even though the transcript level was tested for only a subset of proteins, the transcriptome poorly reflects the proteome, which is in agreement with other studies (Jogaiah *et al.*, 2013; Bai *et al.*, 2015; Walley *et al.*, 2016).

### Early (30 min) mannitol-triggered effects on the leaf phosphoproteome

In the phosphoproteome analysis, 96 unique and 76 significantly differentially abundant phosphosites (mainly derived from phosphopeptides with one phosphorylated residue) were identified (Fig. 1; Supplementary Table S11). Note that problems may arise with peptides containing multiple residues that can be phosphorylated. For instance, an apparent reduced phosphorylation of a singly phosphorylated form of such a peptide may in fact be an increased phosphorylation of another residue within that singly phosphorylated peptide, giving rise to a peptide containing multiple phosphorylated residues that is not picked up by mass spectrometers.

GO analysis of biological process terms revealed that proteins with differentially regulated phosphopeptides under mild osmotic stress (including unique phosphopeptides) were involved in (protein) phosphorylation, regulation of cellular response to stress, response to ABA, and mannitol metabolic process (Supplementary Table S13). To unravel possible interactions between the proteins that were differentially regulated, we constructed, as for the proteome data sets, a functional protein association network consisting of 44 interacting phosphoproteins with 42 (potential) interactions combined with GO categorization for biological processes (see the Materials and methods for details). This approach revealed that interacting phosphoproteins were mainly involved in photosynthesis, splicing, chromatin remodelling, and transport (Supplementary Fig. S3).

Among the differentially regulated phosphoproteins, we identified nine proteins with multiple phosphorylation sites (Table 1). Of these, two phosphoproteins demonstrated a combination of up- and down-regulation, namely CYTOSOLIC INVERTASE 1 (CINV1, AT1G35580) and IQ-DOMAIN 2 (IQD2, AT5G03040). For CINV1, Ser66 and Thr70 belonged to a diphosphorylated peptide and were down-regulated, while Ser73 and Ser547 were up-regulated. In addition, for IQD2, two phosphopeptides were identified of which the one containing phosphorylated Thr339 was up-regulated and the other one containing phosphorylated Ser363 was down-regulated. Multiplicity of phosphorylation and/or dephosphorylation of the same protein could function as a fast, regulatory switch between signalling cascades and metabolic pathways, but were not frequently observed in our data set.

**Table 1.** Phosphorylated proteins with multiple phosphopeptides/sites and their trend in the phosphoproteome data set after 30 min of mannitol treatment

Protein	Name	Description	Position	Group	Log2 fold change
AT1G19870	IQ-DOMAIN 32 (IQD32)	A microtubule-associated protein	Ser459	Down-regulated	–1.332
			Ser479	Unique for control	–
AT1G35580	ALKALINE/NEUTRAL INVERTASE G (A/N-InvG); CYTOSOLIC INVERTASE 1 (CINV1)	Cytosolic invertase that specifically cleaves sucrose into glucose and fructose	Ser66	Down-regulated	–0.693
			Thr70	Down-regulated	–0.693
			Ser73	Up-regulated	0.305
			Ser547	Up-regulated	0.370
AT1G67230	CROWDED NUCLEI 1 (CRWN1); (JAPANESE FOR NUCLEUS) 2 (KAKU2); LITTLE NUCLEI1 (LINC1)	Required for nucleus structure organization (e.g. size and shape)	Ser1001	Up-regulated	0.385
			Ser1027	Up-regulated	0.584
			Ser1093	Unique for mannitol	–
AT2G45820	REMORIN 1.3 (REM1.3)	Remorin family protein	Thr58	Down-regulated	–0.460
			Ser64	Down-regulated	–0.450
AT3G57300	INO80 ORTHOLOG (INO80)	Functions as a positive regulator of DNA homologous recombination (HR) and plays a crucial role in genome stability maintenance	Thr223	Unique for control	–
			Ser228	Unique for control	–
AT4G31880	(ATPDS5C); (PDS5C)	One of five PO76/PDS5 cohesion cofactor orthologs of Arabidopsis. Transcriptional regulator	Thr289	Up-regulated	0.296
			Ser436	Unique for mannitol	–
			Ser559	Unique for mannitol	–
AT4G38550		Phospholipase-like protein (PEARLI 4) family protein	Ser41	Unique for mannitol	–
			Ser153	Up-regulated	0.406
			Thr272	Unique for mannitol	–
AT5G03040	IQ-DOMAIN 2 (IQD2)	IQ-domain 2	Thr339	Up-regulated	0.512
			Ser363	Down-regulated	–0.123
AT5G64270		Splicing factor	Thr230	Up-regulated	0.822
			Thr238	Up-regulated	0.822

Upon mild osmotic stress, the DELLA proteins play an important role in the growth inhibition in leaves upon mild osmotic stress (Claeys *et al.*, 2012). In our phosphoproteome data set, we found a few differentially phosphorylated proteins that are linked to DELLA proteins. For example, BASIC REGION/LEUCINE ZIPPER TRANSCRIPTION FACTOR 16 (bZIP16, AT2G35530; Ser152, >1.5-fold down-regulated) (Table 2), a transcriptional repressor, has been shown to directly repress *REPRESSOR OF GA-LIKE 2* (*RGL2*), encoding a DELLA protein (Hsieh *et al.*, 2012). Another example is the CALCIUM-DEPENDENT PROTEIN KINASE (CDPK/CPK)-RELATED PROTEIN KINASE 2 (TAGK2/CRK2, AT3G19100), for which a phosphosite at Ser57 was up-regulated 1.6-fold upon mannitol treatment (Table 2).

Considering the interest in early signalling cascades underlying responses to mild osmotic stress, we focused on proteins with differentially regulated phosphopeptides possessing protein kinase activity. Using the HMMER online tool (Finn *et al.*, 2015), we found 7 and 10 (potential) protein kinases of which phosphopeptides were up- and down-regulated, respectively (Supplementary Table S15). Some examples of the identified kinases that have a changed phosphostatus upon mild mannitol stress are CALCINEURIN B-LIKE PROTEIN (CBL)-INTERACTING PROTEIN KINASE 8 (CIPK8, AT4G24400), MAP KINASE KINASE KINASE 7 (MKKK7, AT3G13530), BRASSINOSTEROID-SIGNALLING KINASE 1 (BSK1, AT4G35230), and SUCROSE NON-FERMENTING-1 (SNF1)-RELATED PROTEIN KINASE 2.4 (SnRK2.4, AT1G10940) (Table 2). Next, we looked into predicted over-represented kinase motifs as the sequence consensus of phosphopeptide motifs reflects the kinase-specific regulation of substrate phosphorylation and the identity of the corresponding kinases (Supplementary Fig. S4). For the up-regulated phosphopetides, the most enriched motifs were [SP] and [TP], which are targets for AGC kinases, SnRK1, SnRK2, CDPKs, CYCLIN-DEPENDENT KINASEs (CDKs), MITOGEN-ACTIVATED PROTEIN KINASEs (MPKs), RECEPTOR-LIKE KINASEs (RLKs), and SHAGGY-LIKE KINASEs (SLKs) (van Wijk *et al.*, 2014). For the down-regulated phosphopeptides, [SP] and [RxxS] motifs were over-represented, of which the [RxxS] motif is targeted by CDPKs and SnRKs (Kobayashi *et al.*, 2005; Furihata *et al.*, 2006; Vlad *et al.*, 2008).

**Table 2.** Phosphorylated peptides/sites mentioned in the manuscript or on a figure.

Group	Protein	Name (TAIR)	Position	*Log2* fold change	Localisation probability	Peptide with localisation probabilities
Up-regulated	AT1G28280	MPK3/6-TARGETED VQ MOTIF-CONTAINING PROTEIN 1 (MVQ1)	Ser194	0.618	0.95	S(0.018)GS(0.018)S(0.018)NQS(0.945)PNELAAEEK
	AT1G35580	ALKALINE/NEUTRAL INVERTASE G (A/N-InvG); CYTOSOLIC INVERTASE 1 (CINV1)	Ser73	0.305	0.86	SVLDTPLS(0.861)S(0.139)AR
			Ser547	0.370	0.83	RS(0.174)AS(0.826)WPQL
	AT2G21230	BASIC LEUCINE-ZIPPER 30 (bZIP30)	Ser176	0.665	1.00	SIS(1)GEDTSDWSNLVK
	AT3G13290	VARICOSE-RELATED PROTEIN (VCR)	Ser680	0.552	0.99	T(0.005)S(0.005)S(0.99)ADY(0.001)FYVR
	AT3G16830	TOPLESS-RELATED 2 (TPR2)	Thr214	0.399	1.00	ALT(1)PVNLPVAAVAR
	AT3G19100	CALCIUM-DEPENDENT PROTEIN KINASE (CDPK/ CPK)-RELATED PROTEIN KINASE 2 (TAGK2/CRK2)	Ser57	0.683	0.95	ASPFFPFY(0.02)T(0.03)PS(0.95)PAR
	AT4G30190	H(+)-ATPASE 2 (AHA2)	Thr881	0.688	1.00	T(1)LHGLQPK
	AT5G03040	IQ-DOMAIN 2 (IQD2)	Thr339	0.512	1.00	STISVLS(1)ER
Down- regulated	AT1G35580	ALKALINE/NEUTRAL INVERTASE G (A/N-InvG); CYTOSOLIC INVERTASE 1 (CINV1)	Ser66	-0.693	1.00	GRS(1)VLDT(0.984)PLS(0.008)S(0.009)AR
			Thr70	-0.693	1.00	SVLDT(1)PLSSAR
	AT2G35530	BASIC REGION/LEUCINE ZIPPER TRANSCRIPTION FACTOR 16 (bZIP16)	Ser152	-0.708	1.00	GS(1)LGSLNMITGK
	AT2G45820	REMORIN 1.3 (REM1.3)	Thr58	-0.460	1.00	ALAVVEKPIEEHT(1)PK
			Ser64	-0.450	0.95	AS(0.01)S(0.95)GS(0.04)ADRDVILADLEK
	AT2G46020	BRAHMA (BRM); CHROMATIN REMODELING 2 (CHR2)	Ser1762	-0.337	0.83	S(0.174)GS(0.826)WAHDRDEGDEEQVLQPTIK
	AT3G13530	MITOGEN-ACTIVATED PROTEIN KINASE KINASE KINASE 7 (MAPKKK7)	Ser776	-0.274	0.95	LAS(0.051)IS(0.95)GGLDGQAPR
	AT5G03040	IQ-DOMAIN 2 (IQD2)	Ser363	–0.123	1.00	STISVLS(1)ER
Unique for mannitol	AT3G12280	RETINOBLASTOMA- RELATED 1 (RBR1)	Thr406	-	1.00	LAAT(0.999)PVSTAMTTAK
	AT3G58640	Mitogen activated protein kinase kinase kinase-like protein	Ser366	-	0.79	T(0.001)AS(0.028)S(0.182)S(0.789)PEHLSFR
	AT4G24400	CBL-INTERACTING PROTEIN KINASE 8 (CIPK8); SNF1-RELATED PROTEIN KINASE 3.13 (SnRK3.13)	Thr166	-	0.85	T(0.85)T(0.15)CGTPNYVAPEVLSHK
Unique for control	AT1G10940	SNF1-RELATED PROTEIN KINASE 2.4 (SNRK2.4)	Ser357	-	1.00	EVHAS(1)GEVR
	AT4G35230	BRASSINOSTEROID- SIGNALING KINASE 1 (BSK1)	Thr353	-	0.80	KQEEAPS(0.201)T(0.798)PQRPLS(0.001)PLGEACSR
	AT5G40450	REGULATOR OF BULB BIOGENESIS (RBB1)	Ser2802	-	1.00	S(0.997)LS(0.003)DHIQK

### A normalized early mannitol-triggered differential leaf phosphoproteome

**Fig. 5.**
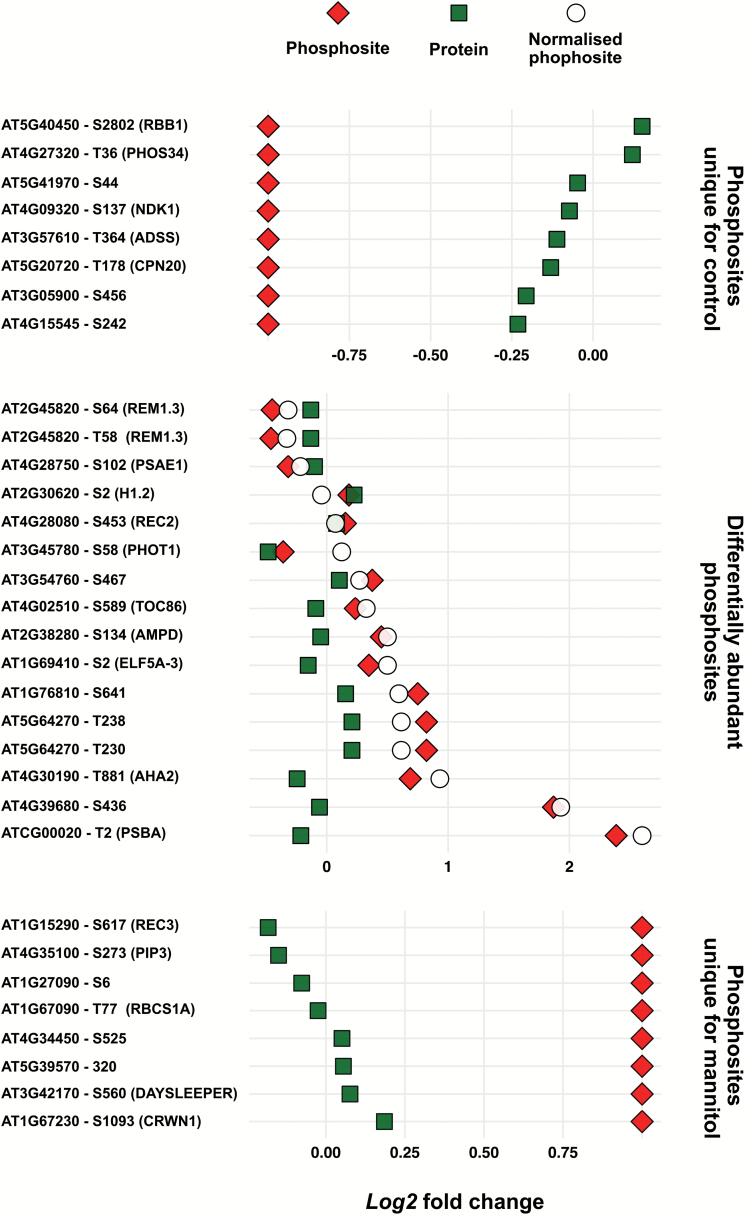
A normalized mannitol-triggered phosphoproteome. Significantly up- and down-regulated phosphopeptides were normalized by subtracting the log2 fold change of the protein abundance from the log2 fold change of the phosphopeptide, with the exception of the unique phosphopeptides. In total, 32 differentially phosphorylated proteins could be mapped on the total proteome data. The differential protein abundance, phosphorylation, and normalized phosphorylation are presented. Unique phosphopeptides for the control and mannitol-treated samples are indicated at log2 fold change –1 and 1, respectively. RBB1, REGULATOR OF BULB BIOGENESIS1; PHOS34, PHOSPHORYLATED PROTEIN OF 34 kDa; NDK1, NUCLEOSIDE DIPHOSPHATE KINASE 1; ADSS, ADENYLOSUCCINATE SYNTHASE; CPN20, CHAPERONIN 20; REM1.3, REMORIN 1.3; PSAE1, PHOTOSYSTEM I REACTION CENTER SUBUNIT IV A; H1.2, HISTONE 1.2; REC2, REDUCED CHLOROPLAST COVERAGE 2; PHOT1, PHOTOTROPIN 1; TOC86, TRANSLOCON AT THE OUTER ENVELOPE MEMBRANE OF CHLOROPLASTS 86; AMPD, ADENOSINE 5'MONOPHOSPHATE DEAMINASE; ELF5A-3, EUKARYOTIC ELONGATION FACTOR 5A-3; AHA2, Hi-ATPASE 2; PSBA, PHOTOSYSTEM II REACTION CENTER PROTEIN A; REC3, REDUCED CHLOROPLAST COVERAGE 3; PIP3, PLASMA MEMBRANE INTRINSIC PROTEIN 3; RBCS1A, RIBULOSE BISPHOSPHATE CARBOXYLASE SMALL CHAIN 1A; DAYSLEEPER, ZINC FINGER BED DOMAIN-CONTAINING PROTEIN; CRWN1, CROWDED NUCLEI 1.

A common question related to quantitative phosphoproteomics is whether the measured phosphorylation changes result from changes in kinase or phosphatase activity or from changes in phosphoprotein abundance (Vu *et al.*, 2016). For the phosphopeptides for which the proteins were detected in the whole proteomic analysis, we normalized phosphopeptide intensity to protein abundance. A total of 158 phosphoproteins (representing the 172 differentially regulated phosphosites) were mapped on the proteome data set and 32 proteins were found to be overlapping. Sixteen phosphopeptides could not be normalized as they belonged to a group of unique phosphopeptides and did not have a fold change value. Next, the log2 fold change of the protein was subtracted from the log2 fold change of the phosphopeptide. This defined a set of phosphorylation events that are fully due to changes in kinase and phosphatase activity and not due to changes in protein abundance (to the extent that these changes in phosphorylation status do not impact on protein abundance) (Fig. 5; Supplementary Table S16). Two phosphopeptides of the remorin family member REM1.3 (AT2G45820), containing phosphorylated Thr58 or Ser64, respectively, were down-regulated upon mannitol treatment (Fig. 5; Table 1). REM1.3 was already reported to be phosphorylated upon oligogalacturonide treatment which elicits a plant stress response (Kohorn *et al.*, 2016). REM1.3 is proposed as a scaffolding protein for signalling at the plasma membrane and is thus an interesting candidate for mannitol-induced signalling (Marín *et al.*, 2012). Additionally, we mapped the whole phosphoproteome, including non-regulated phosphosites, on the whole proteome to identify phosphorylation events that were not identified as significant and thus possibly masked as a result of a difference in protein abundance. Out of the whole data set, 285 phosphosites (corresponding to 173 phosphorylated proteins) could be normalized for their protein abundance and statistical analysis revealed 15 significantly regulated phosphopeptides (Supplementary Table S16). Such an approach allowed us to identify 12 potentially interesting phosphopeptides that were not detected as differentially abundant without a normalization procedure.

### Comparative phosphoproteome analysis identifies bZIP30 and RBB1 as general players in osmotic stress response

**Fig. 6.**
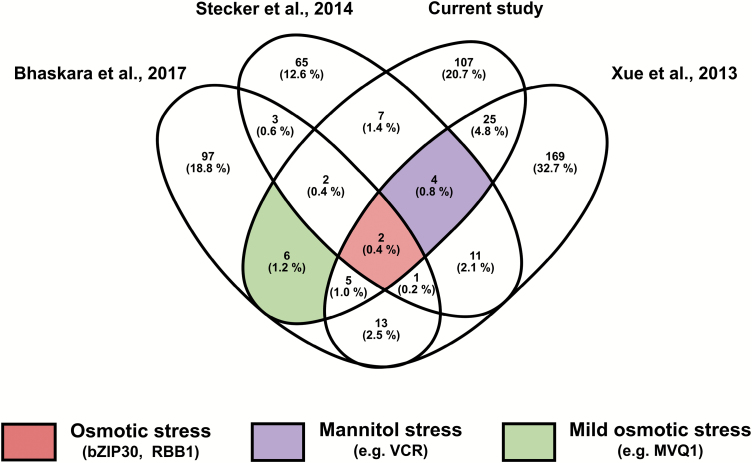
Venn diagram showing the overlapping phosphoproteins from four recent phosphoproteomic studies of osmotic stress responses, including the present study. Details of the experimental set-up of selected studies are indicated in Table 3. bZIP30, BASIC LEUCINE-ZIPPER 30; RBB1, REGULATOR OF BULB BIOGENESIS1; VCR, VARICOSE-RELATED PROTEIN; MVQ1, MPK3/6-TARGETED VQ MOTIF-CONTAINING PROTEIN 1.

To assess whether the mannitol-regulated phosphorylated proteins in growing leaf tissue are part of a more general stress response or are specific for the stress-induced growth-regulating response, we compared our data set (25 mM mannitol for 30 min) with three previously published osmotic stress-related phosphoproteome data sets (Xue *et al.*, 2013; Stecker *et al.*, 2014; Bhaskara *et al.*, 2017*b*). Given the differences in experimental set-up (long-term versus short-term or mild versus severe stress) (see Table 3 for details), we found little overlap between all four data sets; only two proteins, BASIC LEUCINE-ZIPPER 30 (BZIP30)/DRINK ME (DKM) and REGULATOR OF BULB BIOGENESIS1 (RBB1) (Han *et al.*, 2015), were detected in all data sets, possibly indicating a general role in osmotic stress responses (Fig. 6; Supplementary Table S17). Interestingly, a phosphopeptide with the same Ser176 phosphorylation site as bZIP30 was found to be up-regulated in three data sets where mannitol was used. In addition, we found four common mannitol-responsive phosphoproteins. Six phosphoproteins were exclusively found in the two mild osmotic stress studies, including a MITOGEN ACTIVATED PROTEIN KINASE KINASE KINASE-LIKE protein (AT3G58640) and MPK3/6-TARGETED VQ MOTIF-CONTAINING PROTEIN 1 (MVQ1, AT1G28280).

**Table 3.** Details on the four phosphoproteomic studies used for comparative analysis

Study	Material	Growth medium	Compound	Concentration	Time	Setup	Method
Current study	Leaf #3 of 15-day-old seedlings	Agar plates	Mannitol	25 mM	30 min	Transfer	Label-free
Bhaskara *et al*. (2017b)	7-day-old seedlings	Agar plates	PEG	Low water potential (–1.2 MPa)	96 h	Transfer	iTRAQ
Stecker *et al.* (2014)	10-day-old seedlings	Liquid culture	Mannitol	300 mM	5 min	Medium replacement	^15^N-labelling
Xue *et al.* (2013)	12-day-old seedlings	Liquid culture	Mannitol	800 mM	30 min	Transfer	Label-free

PEG, polyethylene glycol; iTRAQ, isobaric tags for relative and absolute quantitation.

### (Phospho) proteome profiling identifies AHA2 and CRRSP38 as potential leaf growth regulators

**Fig. 7.**
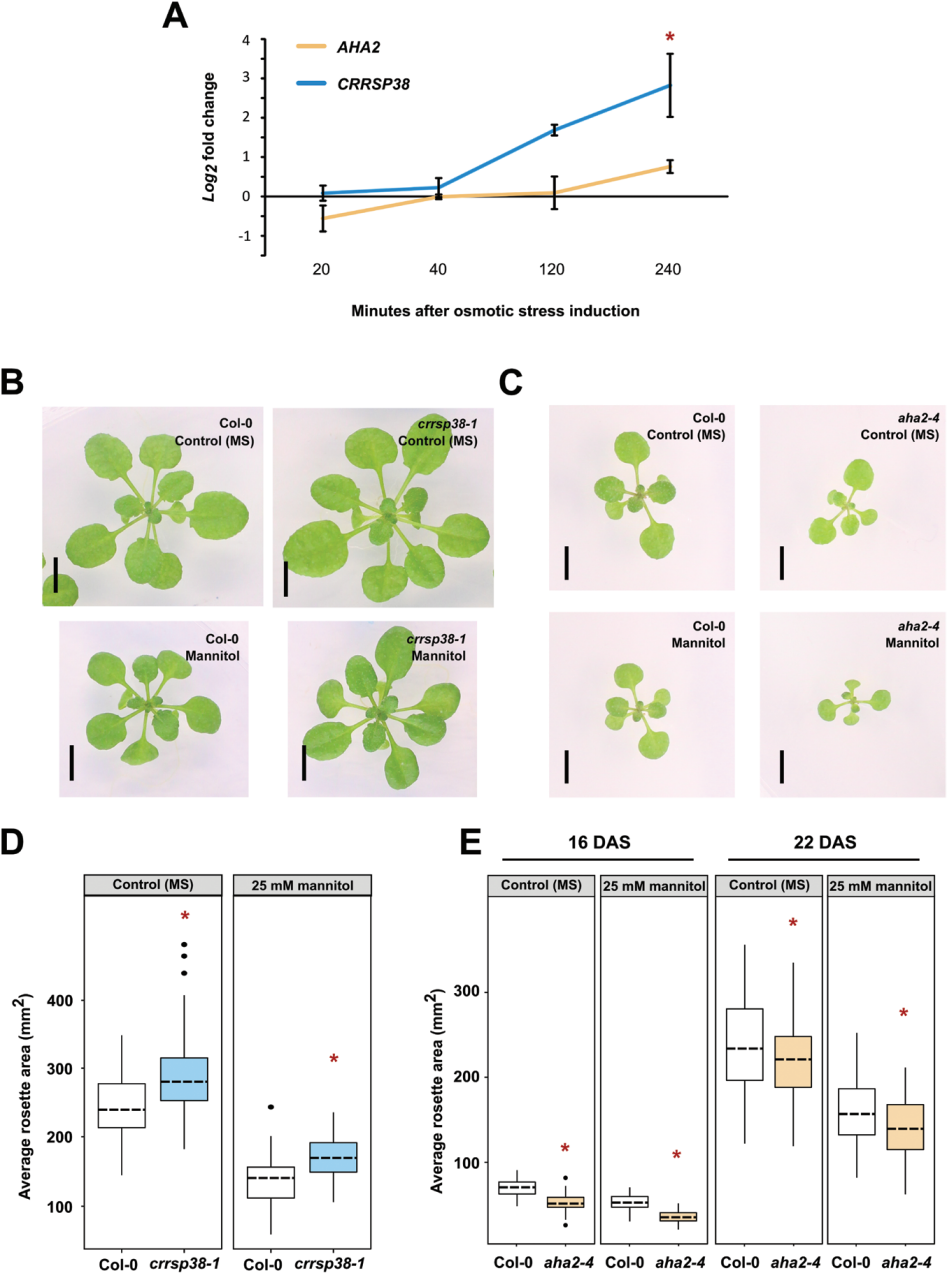
Phenotypic analysis of T-DNA insertion lines for selected candidates. (A) Expression of *AHA2* and *CRRSP38* upon mannitol-induced osmotic stress. Error bars represent the SEs. Statistical significance (Student’s *t*-test), comparing mannitol-treated and control (MS) samples, is indicated: *P<0.05. (B–E) Leaf growth phenotype of *crrsp38-1* and *aha2-4*. (B, C) Representative pictures of *crrsp38-1* (22 DAS) (B) and *aha2-4* (16 DAS) (C) rosettes compared to the wild type (Col-0) grown on control (MS) or mannitol (25 mM)- containing medium. DAS, days after stratification. Scale bar=5 mm. (D, E) Quantification of the rosette area of *crrsp38-1* at 22 DAS (D) and *aha2-4* at 16 and 22 DAS (E) grown on control (MS) or 25 mM mannitol. Boxplots are combined values of at least 80 (22 DAS) or 40 seedlings (16 DAS) from different plates and from four (22 DAS) or two (16 DAS) independent experiments. Statistical significance (Tukey’s test), comparing mutant lines and Col-0, is indicated: *P <0.05.

To assess the involvement of proteins with a differential abundance or phosphorylation status in leaf growth, and thus the quality of our data set, we selected two candidates for phenotypic analysis. From the mannitol-regulated proteome data set (Supplementary Table S10), we selected the poorly characterized CYSTEINE-RICH REPEAT SECRETORY PROTEIN 38 (CRRSP38, AT3G22060). Together with 15 other proteins, CRRSP38 was identified as down-regulated after 30 min of mannitol treatment and was not detected after 4 h of mannitol-induced stress. Transcriptionally, *CRRSP38* did not show any mannitol-induced changes at 20 min and 40 min, and an increasing expression from 2 h onwards (Fig. 7A). We obtained a knock-out allele of *CRRSP38* (SALK_151902), referred to as *crrsp38-1* (Supplementary Fig. S5). Phenotypic analysis of *crrsp38-1* at 22 DAS revealed a larger rosette area of 18% and 26% under control and mild mannitol conditions, respectively (Fig. 7B, D).

From the normalized phosphoproteome, we focused on AHA2 (AT4G30190), an H(+)-ATPASE 2 of which a phosphopeptide containing the Thr881 phosphosite showed, depending on the normalization procedure, an 1.9- or 2.5-fold increase in phosphorylation after mannitol treatment and does not show any obvious mannitol-induced transcriptional changes in a time course experiment (Figs 5, 7A; Supplementary Table S16). Thr881 is situated in the conserved autoinhibitory region I of the C-terminal domain of AHA2 (Rudashevskaya *et al.*, 2012; Falhof *et al.*, 2016), and rapid PLANT PEPTIDE CONTAINING SULFATED TYROSINE 1 (PSY1)-induced *in planta* phosphorylation of AHA2 at Thr881 increases proton efflux (Fuglsang *et al.*, 2014). To assess the role of AHA2 in growth inhibition upon mild osmotic stress, we characterized the strong knock-down *aha2-4* mutant with only ~10% *AHA2* expression compared to the wild type (Haruta *et al.*, 2010). The *aha2-4* mutant showed a significant reduction in rosette area under control and mild mannitol conditions compared to wild-type plants (Fig. 7C, E). This reduction in rosette size is more pronounced at the earlier stages of plant growth (16 DAS) where *aha2-4* mutants have a 25% and 32% reduction in rosette area compared to the wild type under control and mannitol conditions, respectively (Fig. 7C, E). At later stages of plant growth (22 DAS), the difference in rosette size is less pronounced, namely *aha2-4* mutants have an 8% and 11% reduction in rosette area compared to the wild type at 16 DAS under control and mannitol conditions, respectively.

In summary, the above-described results provide preliminary evidence for CRRSP38 as a novel regulator of leaf growth and also assigned a new role to AHA2 during leaf growth.

## Discussion

### Identification of GA-related growth-regulating pathways in the mild osmotic stress response

Several mild osmotic stress regulators have been identified (Dubois *et al.*, 2013; Van den Broeck *et al.*, 2017). Recently, it was shown that in growing leaves upon mild osmotic stress, a transcriptional network of 20 transcription factors is induced and those transcription factors are, at least partially, responsible for the stress-induced growth inhibition (Van den Broeck *et al.*, 2017). The stress-induced network has multiple connections to a GA degradation enzyme, GA2-OX6. The induced GA2-OX6 results in the degradation of bioactive GA which in turn leads to the stabilization of DELLA proteins and the observed growth inhibition. However, in our whole proteome analysis, the network transcription factors, the DELLA proteins or the GA2-OX6 protein were not detected. This is possibly the result of a low coverage of the proteome and/or the limitation of our MS set-up. However, two other phosphoproteins with regulatory links to the GA–DELLA pathway were identified. First, the transcription factor bZIP16 showed a decrease in phosphorylation upon mild osmotic stress. As bZIP16 can repress *RGL2* expression (Hsieh *et al.*, 2012), encoding a DELLA protein, we hypothesize that the dephosphorylation of bZIP16 could reduce its repressing activities on *RGL2*. Interestingly, when exploring bZIP16 in the functional protein association network, bZIP16 interacts with TPR2 (TOPLESS RELATED 2), which in turn is also differentially phosphorylated in our data set and is most probably responsible for the repressing activities of bZIP16. Secondly, TAGK2/CRK2, a kinase that regulates the GA RECEPTOR RING E3 UBIQUITIN LIGASE (GARU) at Tyr321 which results in the disruption of the interaction between GARU and the GA RECEPTOR GA INSENSTIVE DWARF1 (GID1) (Nemoto *et al.*, 2017), showed an increased phosphorylation. However, the role of phosphorylation associated with TAGK2 has not yet been studied. Changes in the kinase activity of TAGK2/CRK2 could thus have an effect on GA levels and subsequent DELLA stabilization. Both differentially phosphorylated proteins could be part of alternative pathways, in addition to the previous unravelled network, that regulate DELLA activity and thus the subsequent growth repression under mild osmotic stress conditions.

### Regulation of the cell cycle and cell expansion

One differentially phosphorylated transcription factor that appears to be an osmotic stress regulator is bZIP30 (Fig. 6). This transcription factor influences the expression of cell cycle and cell expansion genes, two processes that are affected by mild mannitol treatment (Skirycz *et al.*, 2011*b*; Lozano-Sotomayor *et al.*, 2016). Our network construction also indicated that bZIP30 is predicted to interact with BRAHMA, the SWI/SNF chromatin-remodelling ATPase, providing another potential growth-regulatory pathway. Moreover, RBR1 (RETINOBLASTOMA-RELATED1), a key cell cycle-related protein, is differentially phosphorylated under mild osmotic stress at Tyr406, positioned in the A-domain of RBR1 enabling protein interactions (Gibson *et al.*, 1994; Lee *et al.*, 1998). As we studied expanding leaves, we hypothesize that the phosphorylation of RBR1 upon mannitol release disrupts the interaction with other proteins, such as E2Fa, and might push proliferating cells into premature differentiation (Magyar *et al.*, 2012; Polyn *et al.*, 2015).

### Proteins involved in translation, oxidation–reduction, photosynthesis, and carbohydrate metabolism are affected upon mild osmotic stress

Three main biological processes are affected in the proteome of growing leaves exposed to mild osmotic stress. First, ribosomal proteins were highly regulated upon mannitol exposure, suggesting an altered capacity for protein translation. At 30 min of mannitol treatment, the translational machinery was both up- and down-regulated while at 4 h translation was mainly down-regulated. This is in line with the observation of long-term proteome changes in growing *A. thaliana* leaves subjected to mannitol, where the levels of ribosomal and translational proteins were also found to be highly regulated (Skirycz *et al.*, 2011*b*).

Our data indicated down-regulation of oxidation–reduction proteins in growing leaves exposed to mild osmotic stress. This is in contrast to the long-term proteome changes in growing leaf tissue (Skirycz *et al.*, 2011*b*) and not expected because peroxidases have been widely described as ROS scavengers and are involved in repair of ROS damage (Kapoor and Sveenivasan, 1988; Caverzan *et al.*, 2012; Bela *et al.*, 2015). However, on a transcript level, glutathione peroxidases have already been shown to be down-regulated in rice upon drought stress (Passaia *et al.*, 2013). It is known that ROS play a dual role, being toxic and destructive molecules, but they can also serve as signalling molecules regulating stress responses, growth, and development (Kovtun *et al.*, 2000; Foyer and Noctor, 2005; Pitzschke and Hirt, 2006; Hossain *et al.*, 2015). In addition, the down-regulation of scavenger proteins might be a result of the lack of ROS production during the very early stages of mild osmotic stress or because these enzymes are a source of hydrogen peroxide (H_2_O_2_) (Blokhina *et al.*, 2003; Beffagna *et al.*, 2005). Deficiency in some ROS scavengers led to an increase in the plant’s sensitivity to oxidative stress, while exposure to osmotic and salt stresses resulted in an increased tolerance (Miller *et al.*, 2007).

Together with oxidation–reduction proteins, proteins involved in photosynthesis and carbohydrate metabolism were also down-regulated under 30 min of mild mannitol stress. The only protein that was up-regulated upon mannitol treatment was PEPC. This is in agreement with previous studies where it was shown that abiotic stresses can induce *PEPC* gene expression in wheat, *A. thaliana*, and sorghum (Echevarría *et al.*, 2001; González *et al.*, 2003; García-Mauriño *et al.*, 2003; Sánchez *et al.*, 2006). Moreover, recent studies demonstrated that the maize *PEPC* gene was able to confer drought tolerance and increase grain yield in transgenic wheat plants (Qin *et al.*, 2016). Furthermore, in agreement with an earlier report on drought (Lawlor and Tezara, 2009), our data showed that photosynthesis-related proteins, such as PHOTOSYSTEM II SUBUNIT Q and PHOTOSYSTEM II SUBUNIT P, were down-regulated upon mannitol-induced stress, as were many chloroplast-located proteins. This was expected as the photosynthetic electron transfer chain can produce ROS (Ramachandra Reddy *et al.*, 2004).

### Mannitol-mediated (phospho)proteome can identify novel growth-regulating proteins

We explored the role of CRRSP38, a member of the large family of CRRSPs containing at least 60 members in *A. thaliana* (Chen, 2001). Although CRRSP38 is poorly characterized, there is some indication for roles in jasmonic acid, ABA, and salt stress responses and regulation of iron acquisition in *A. thaliana* and rice (Xin *et al.*, 2005; Jiang *et al.*, 2007; Zhang *et al.*, 2009; Serra *et al.*, 2013; Yang *et al.*, 2013). The CRRSP38 protein level was down-regulated in growing leaves exposed to 30 min of mannitol-induced stress. Although no transcriptional changes were detected in the first 40 min after mild mannitol treatment, the expression of *CRRSP38* went gradually up from 2 h onwards. As CRRSP38 was not detected in our proteome data set after 4 h, no conclusion can be drawn on the protein abundance of CRRSP38 upon a more long-term mannitol exposure. A likely loss-of-function line of *CRRSP38* showed an increased rosette size under both control and mild mannitol conditions compared to the wild type. However, it should be noted that due to the lack of a second allele and complementation data (Nikonorova *et al.*, 2018*b*), these results are only indicative and, to demonstrate conclusively a role for CRRSP38 in (stress-regulated) leaf growth, more work is required. Nevertheless, *CRRSP38* is also differentially expressed during early *A. thaliana* leaf growth (Andriankaja *et al.*, 2012), further indicating a potential role in this process.

Finally, the importance of the plasma membrane H^+^-ATPase for plant growth and its tight regulation via phosphorylation was established in the past decades (Haruta *et al.*, 2010, 2018; Falhof *et al.*, 2016). AHA2, a H^+^-ATPase, was transcriptionally not regulated or differentially abundant in the short-term proteome data set but purely post-translationally regulated. In our study, an AHA2 phosphopeptide containing phosphorylated Thr881 was up-regulated after 30 min of mild mannitol treatment. This phosphosite is located in the C-terminal cytoplasmic domain and important for its activity (Niittylä *et al.*, 2007). Moreover, a direct link between growth regulation and pump activation via phosphorylation of Thr881 was already shown (Fuglsang *et al.*, 2014). Our phenotypic data showed that the *aha2-4* mutant resulted in smaller rosettes under control conditions and mild mannitol stress.

The lack of a sensitivity phenotype towards mild mannitol stress of *crrsp38-1* and *aha2-4* suggests that CRRSP38 and AHA2 could be general growth-regulating proteins. Because we sampled leaves whose growth is being repressed as a result of the mild mannitol treatment, both growth-regulating and stress response-regulating proteins can be detected. However, these results are only indicative, and more work is required to add CRRSP38 and AHA2 to the (stress-regulated) leaf growth pathway. It will be interesting to explore the molecular function of CRRSP38 and AHA2 in leaf growth regulation, and its precise role in regulating growth upon mild mannitol-induced stress.

### Conclusions

In this study, label-free proteomic and phosphoproteomic analyses were performed on expanding *A. thaliana* leaves exposed to mild osmotic stress. By performing the proteome analysis at two different time points, 30 min and 4 h, dynamic patterns in protein abundance could be observed. In general, after 30 min of stress, ribosomal proteins were up-regulated upon mannitol treatment, and photosynthesis and reduction–oxidation-related proteins were down-regulated; while, after 4 h, ribosomal proteins were down-regulated. Furthermore, the lack of correlation between transcriptional changes prior to changes in protein abundance points towards an important role for protein degradation/stabilization upon stress. In addition, we identified several proteins that had an altered phosphorylation status upon mild osmotic stress, suggesting an important role for kinase- and phosphatase-mediated signalling. We also identified several important regulators, such as the transcription factor bZIP30, which is probably a central component of both mild and severe osmotic stress. However, previously and in this study, no transcriptional changes were observed for bZIP30, indicating that by solely studying transcriptomics, central proteins involved in stress response are likely to be missed. On the other hand, several transcription factors from the recently described mild mannitol stress-associated gene regulatory network were not identified in our (phospho)proteome data sets. It should be noted that this could also be the result of the lack of full coverage of the (phospho)proteome. In addition, we identified proteins that were phosphorylated under short-term mild osmotic stress, such as AHA2. Phenotypic analysis of an *aha2-4* knock-out mutant indeed confirmed a role for AHA2 in the regulation of leaf growth.

Taken together, our data sets further stress the importance of proteome- and phosphoproteome-based approaches, in addition to transcriptomics, for unravelling the molecular mechanisms underlying growth regulation under stress. To pinpoint candidate phosphorylated proteins and specific phosphosites for further analysis, our data sets can also be easily consulted through our PTMViewer (bioinformatics.psb.ugent.be/webtools/ptm_viewer/).

## Supplementary data

Supplementary data are available at *JXB* online.

Fig. S1. Visual explanation of the three subsets described in the main text.

Fig. S2. Functional protein association network of significant mannitol-regulated proteins (4 h treatment).

Fig. S3. Functional protein association network of significant mannitol-regulated phosphopeptides mapped on the corresponding proteins (30 min treatment).

Fig. S4. Visual depiction of predicted over-represented kinase motifs based on Motif X analysis.

Fig. S5. Details of the *crrsp38-1* T-DNA line.

Protocol S1. Details on (phospho)proteomics workflows and data analysis.

Table S1. List of primers used for expression analysis after 20 min and 40 min of mannitol treatment.

Table S2. List of primers used for expression analysis after 4 h of mannitol treatment.

Table S3. List of primers used for the genotyping of *aha2-4* and *crrsp38-1* mutant alleles.

Table S4. Output of ANOVA test for *crrsp38-1*.

Table S5. Output of Tukey’s post-hoc test for *crrsp38-1*.

Table S6. Output of ANOVA test for *aha2-4* (16 days after stratification).

Table S7. Output of Tukey’s post-hoc test for *aha2-4* (16 days after stratification).

Table S8. Output of ANOVA test for *aha2-4* (22 days after stratification).

Table S9. Output of Tukey’s post-hoc test for *aha2-4* (22 days after stratification).

Table S10. Proteins identified at 30 min after mannitol treatment, including raw data, differentially abundant and unique proteins.

Table S11. Phosphopeptides identified at 30 min after mannitol treatment, including raw data, differentially abundant and unique phosphopeptides.

Table S12. Proteins identified at 4 h after mannitol treatment, including raw data, differentially abundant and unique proteins.

Table S13. GO enrichment on biological processes.

Table S14. Comparative analysis of proteome data sets from 30 min (significantly different and unique proteins) and 4 h (the complete data set) mannitol treatment.

Table S15. Protein kinases with differentially phosphorylated and unique phosphopeptides predicted by HMMER.

Table S16. Phosphorylated peptides normalized for protein abundance.

Table S17. Comparative analysis of outputs from four phosphoproteomic studies on osmotic stress.

Supplementary Figures S1-S5 and Tables S1-S9

Supplementary Table S10

Supplementary Table S11

Supplementary Table S12

Supplementary Table S13

Supplementary Table S14

Supplementary Table S15

Supplementary Table S16

Supplementary Table S17

## Data deposition

All MS proteomics data have been deposited in the ProteomeXchange Consortium via the PRIDE partner repository with the data set identifier PXD008900.
