# Supplementary material for: Early mannitol-triggered changes in the Arabidopsis leaf (phospho)proteome reveal growth regulators
Source: J Exp Bot. 2018 Jul 13;69(19):4591–607. doi: 10.1093/jxb/ery261 (PMC6117580; doi:10.1093/jxb/ery261)
Supplement: Supplementary Figures S1-S5 and Tables S1-S9 [file ery261_suppl_supplementary_materials.pdf]

# Supplementary Information

## Early mannitol-triggered changes in the Arabidopsis leaf (phospho)proteome reveal growth regulators

Natalia Nikonorova[1,2,*], Lisa Van den Broeck[1,2,*], Shanshuo Zhu[1,2,3,4], Brigitte van de Cotte[1,2,], Marieke Dubois[1,2,$], Kris Gevaert[3,4], Dirk Inzé[1,2], and Ive De Smet[1,2,#]

## SUPPLEMENTARY FIGURES

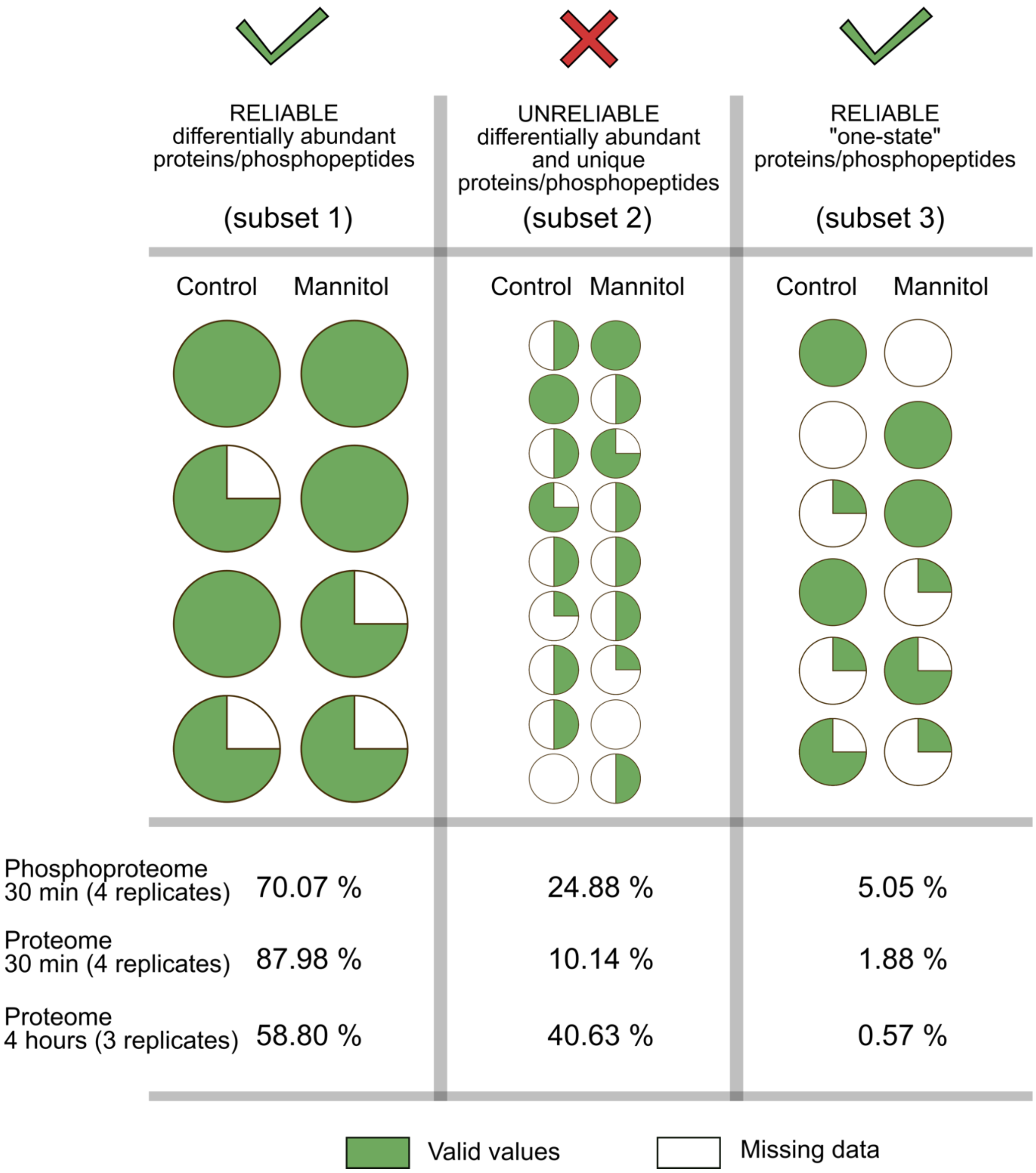


**Figure S1. Visual explanation for the 3 subsets described in the main text.** The percentages for each data set and each subset are indicated. The values detected for a protein or phosphosite were visualized in a circle and each quarter of a circle represents a biological repeat. For example, a half green circle means that for only 2 repeats valid values were detected. Each protein/phosphosite was subdivided into three subsets depending on the percentage of valid values.

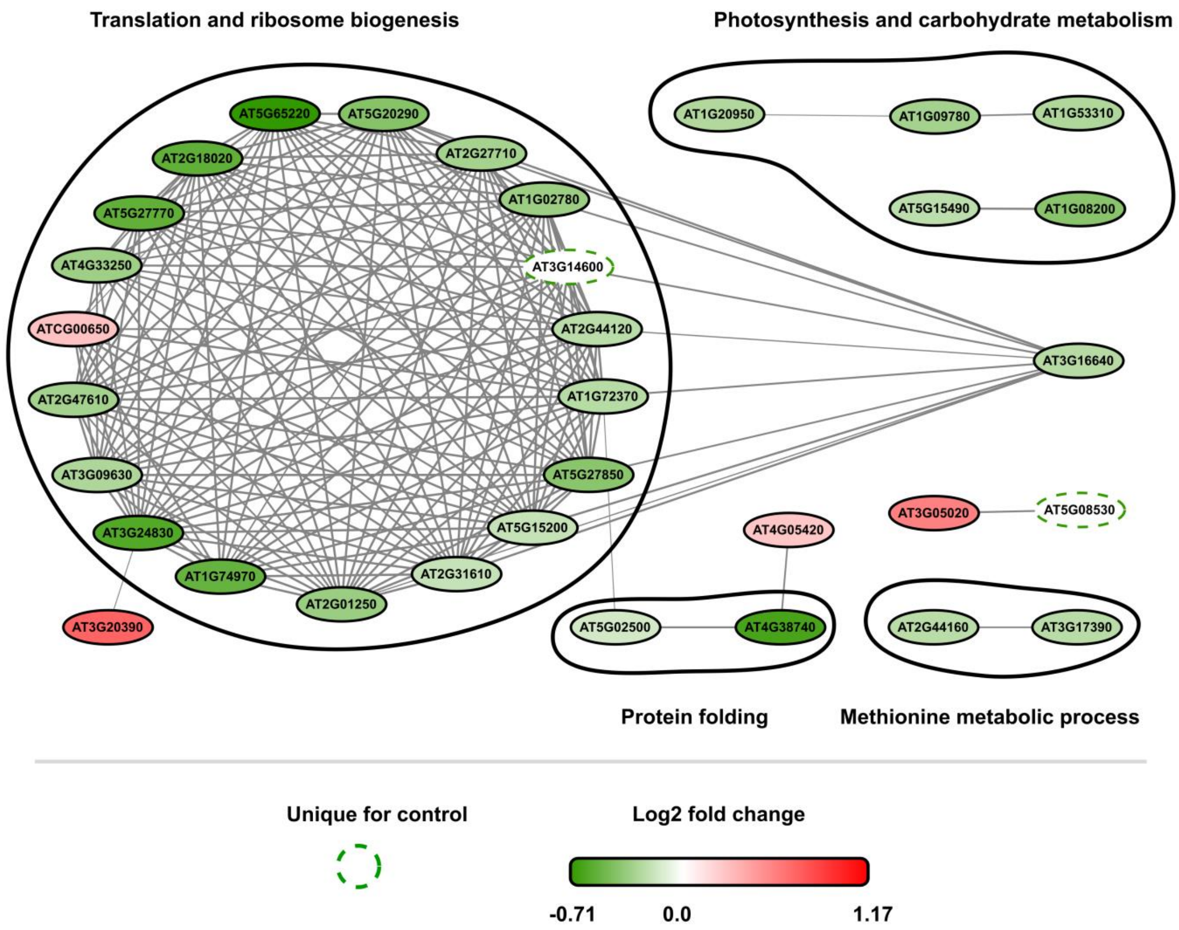


**Figure S2. Functional protein association network of significant mannitol-regulated proteins (4 h treatment).** GO annotations for biological process of up- and downregulated proteins were superimposed on the network and nodes were grouped accordingly. Unique proteins were indicated with dashed lines while differentially abundant proteins were coloured from dark green ranging to red depending on the $log_2$ fold change. Thickness of connecting lines indicates a combined score of interaction.

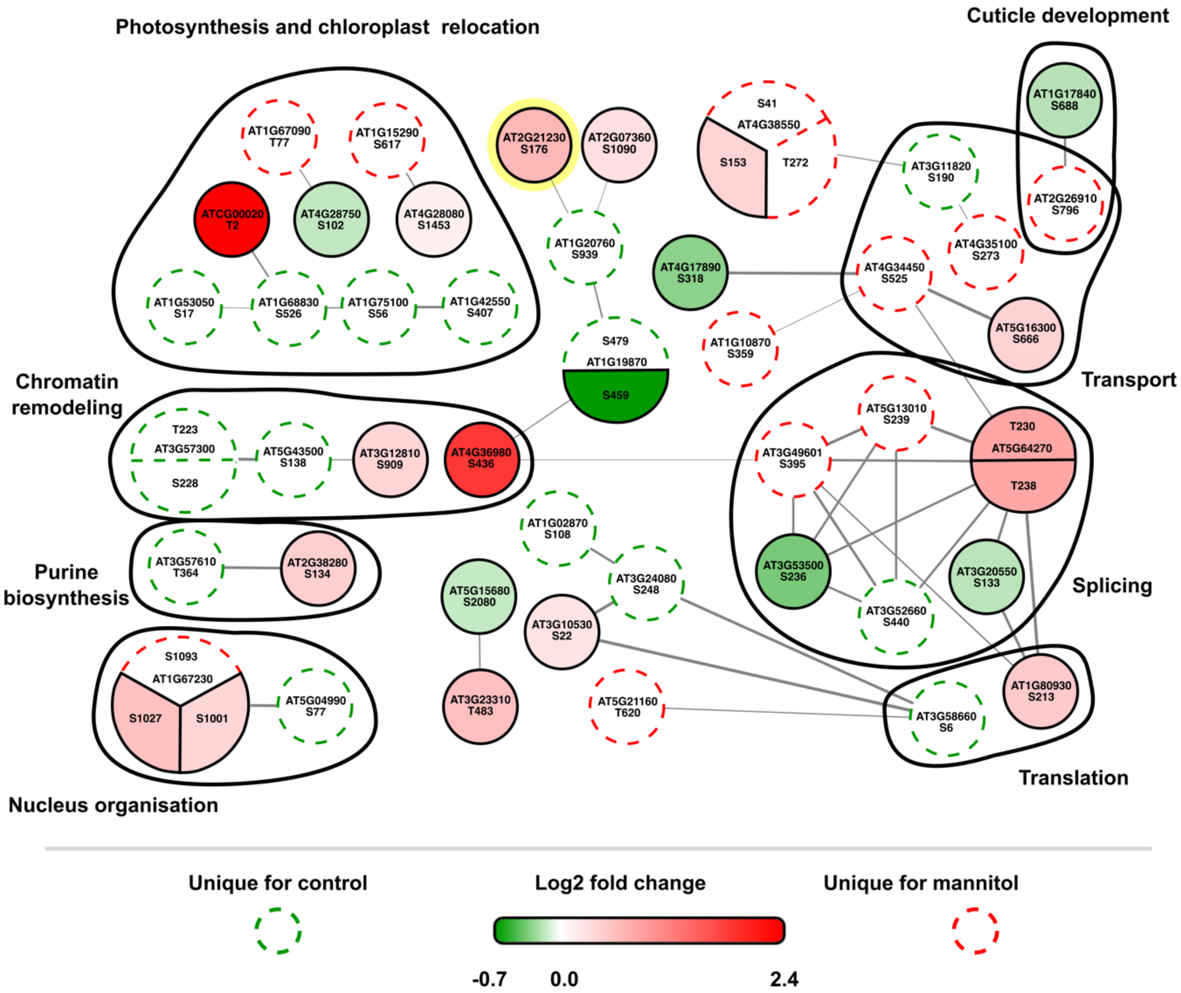


**Figure S3. Functional protein association network of significant mannitol-regulated phosphopeptides mapped on the corresponding proteins (30 min treatment).** GO annotations for biological process of up- and downregulated proteins were superimposed on the network and nodes were grouped accordingly. Unique phosphosites were indicated with dashed lines while differentially abundant phosphosites were coloured from dark green ranging to red depending on the *log*$_2$ fold change. Thickness of connecting lines indicates a combined score of interaction. Transcription factor bZIP30 is highlighted in yellow.

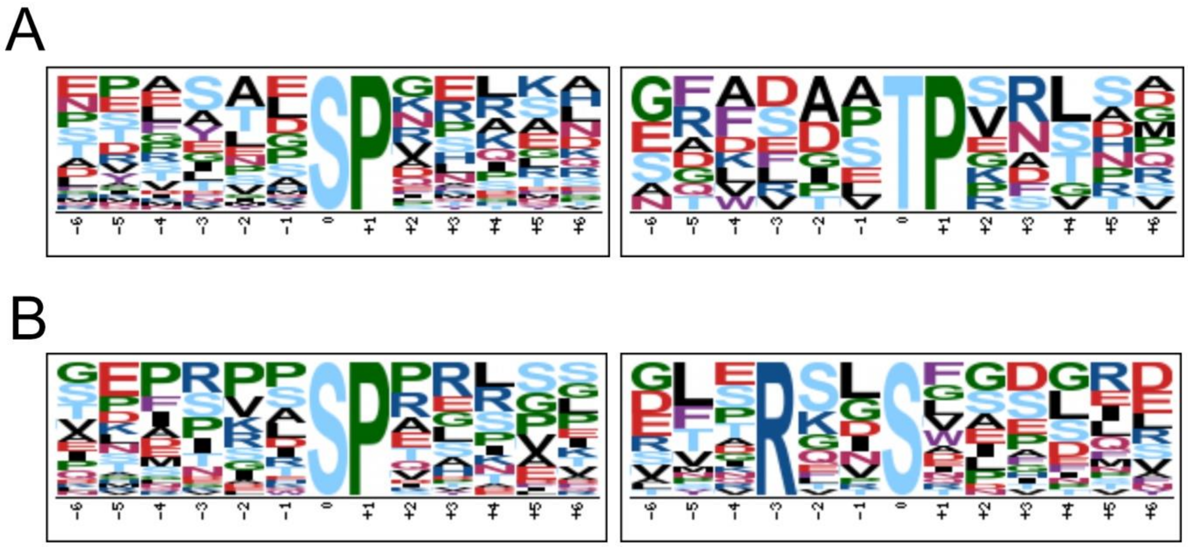

**Figure S4.** Visual depiction of predicted overrepresented kinase motifs based on Motif X analysis for upregulated (A) and downregulated phosphopeptides (B).

A

B

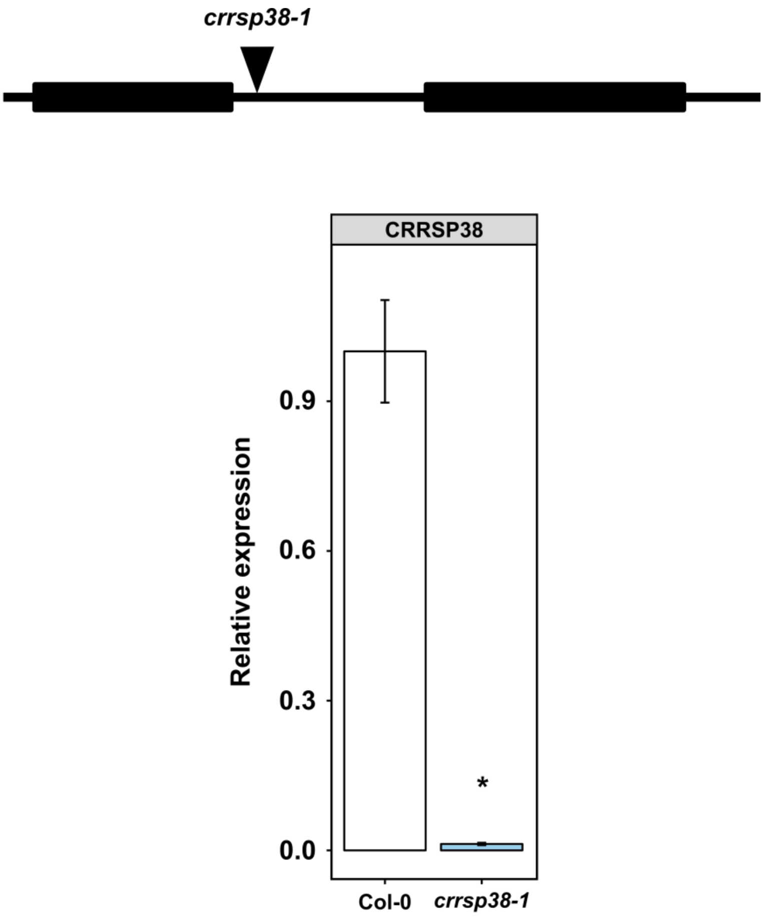


**Figure S5. Details of *crrsp38-1* T-DNA line. (A)** Genomic region of *CRRSP38* indicating exons (black rectangles) and position of T-DNA (triangle) for *crrsp38-1*. **(B)** *CRRSP38* expression in *crrsp38-1* T-DNA line. Statistical significance (Student's *t*-test) compared to Col-0 is indicated: *, p-value < 0.05.

# SUPPLEMENTARY PROTOCOL

## (Phospho)proteome workflow (30 min)

Proteins were extracted in homogenization buffer (pH 8.0) containing 30% sucrose, 250 mM Tris-HCl, 5 mM EDTA, 1 mM DTT and protease and phosphatase inhibitor cocktails (cOmplete™ and PhosSTOP™). The samples were sonicated on ice and centrifuged at 4°C for 15 min at 2500 × *g* to remove debris. Supernatants were collected and a methanol/chloroform precipitation was carried out by adding methanol, chloroform and water in the following proportion 3:1:4. Samples were centrifuged for 10 min at 5000 × *g*, and the aqueous phase was removed. After addition of 4 volumes methanol, the proteins were pelleted via centrifugation for 10 min at 2500 × *g*. Pellets were washed with 80% acetone and re-suspended in 8 M urea in 50 mM triethylammonium bicarbonate (TEAB) buffer (pH 8). Alkylation of cysteines was carried out by adding a combination of tris(carboxyethyl)phosphine (TCEP, Pierce) and iodoacetamide (Sigma-Aldrich) to final concentrations of 15 mM and 30 mM, respectively, and the reaction was allowed for 15 min at 30°C in the dark. Before digestion, the protein concentration was measured with a Nanodrop Spectrophotometer. Two milligrams of the proteins were pre-digested with endoproteinase-LysC (Wako Chemicals) for 2 h, followed by a digestion with trypsin overnight (Promega Trypsin Gold, mass spectrometry grade), both digestions occurring at 37°C at an enzyme-to-substrate ratio of 1:100 (w:w). Prior trypsin digestion samples were diluted 8 times in order to lowered urea concentration. The digest was acidified to pH ≤ 3 with trifluoroacetic acid (TFA), desalted with SampliQ C18 SPE cartridges (Agilent) according to the manufacturer's guidelines and vacuim-dried in a SpeedVac. Peptide pellets were dissolved in 500 µl of 80% acetonitrile (ACN) and 6% TFA. Thirty µl of each peptide solution was used for proteome analysis and the rest for the phosphopeptide enrichment which was performed as previously described (Vu *et al.*, 2016). (Phospho)peptide samples were vacuum-dried and re-dissolved prior to LC-MS/MS analysis in 2% acetonitrile and 0.1% TFA.

## Data filtering approach

To overcome the problem of missing values without imputation or complex statistical analysis, we applied a hybrid approach for data analysis that treats intensity-based and

presence/absence data separately. The original, complete dataset containing *log2*-transformed intensities was split in three subsets (**Figure S1**). The first subset consisted of proteins or phosphopeptides that were quantified in ≥ 75 % of the biological replicates in both control and mannitol-treated samples. These proteins or phosphopeptides were thus detected in at least 3 out of 4 biological replicates (or all biological replicates for the 4 h data set) and had therefore none or few missing values. Only this subset was directly submitted for linear regression analysis, where the few missing values were ignored. The second subset contained proteins or phosphopeptides that were quantified in > 25% and < 75% of the biological replicates of at least one treatment. This group was named "unreliable" as only half of the replicates could be quantified and, was therefore excluded from further analysis. The third subset included proteins or phosphopeptides that were present in one sample (in more than 75 % of the replicates) and absent or below the detection threshold in another sample (in more than 75 % of the replicates). This subset contained unique proteins or phosphopeptides that are quite often incorrectly ignored and excluded from final results. Subsets of unique proteins or phosphopeptides were extracted and used as such, without any subsequent statistical analysis.

**Normalisation of phosphopeptide abundances**

For the phosphosites of which the proteins were detected in the whole proteomic analysis, we normalised phosphopeptide intensity to protein abundance. As a validation of statistically significant phosphosites only $log_2$ fold change was normalised. Namely, the $log_2$ fold change of the protein was subtracted from the $log_2$ fold change of the phosphopeptide.

To identify putative phosphorylation events that were not identified as significant and thus masked as a result of a difference in protein abundance, we mapped the whole phosphoproteome, including non-significant phosphosites, on the whole proteome. Prior normalisation intensity values were $log_2$-transformed and filtered for missing values. Each sample was centred by subtraction of median. Then, the $log_2$-transformed centred intensity value of protein was subtracted from the $log_2$-transformed centred intensity value of the phosphopeptide. Obtained normalised phosphopeptide intensity values were subjected to statistical analysis (Student's T-test, $p < 0.05$).

## Primer lists

**Supplementary Table S1.** List of primers used for expression analysis after 20 and 40 min of mannitol treatment. In addition to the AT-code, gene name, forward and reverse primer, the trend of the protein abundance or phosphorylation event is listed.

| AT-Code | Gene name | Trend | FWD | REV |
|---|---|---|---|---|
| AT5G63980 | SAL1 | Downregulated | TTCTGTATTCGGACGCAAGT | GCAGCTTTCTTAGCAGCATCAA |
| AT3G26060 | PEROXIREDOXIN Q (PRXQ) | Downregulated | GGCTCCACACTCACTCACTC | AACCTTGGCAAAGATTAAGCC |
| AT2G44060 | LEA26 | Downregulated | TTTGGGAAGCCAACTGCTGA | ATCGCTCTCGACCAGGTAGT |
| AT1G76080 | CHLOROPLASTIC DROUGHT-INDUCED STRESS PROTEIN OF 32 KD (CDSP32) | Downregulated | ATCGTGCTTGACGTTGGTCT | GGACATTGACCGGGACAGTT |
| AT4G10450 | 60S RIBOSOMAL PROTEIN L9-2 (RPL9D) | UNIQUE for mannitol | TGATCAATCAGAAATGTCATGTGAA | ACTTCATTCCTCGACTGCGA |
| AT2G26780 | ARM repeat family | UNIQUE for mannitol | GTTCAACCATCCAGGAGGCA | ACTTTATAAACCCCGCCAGAGA |
| AT3G11170 | FATTY ACID DESATURASE 7 (FAD7) | UNIQUE for mannitol | TCACCACCAGAACCATGGAC | AAGCATCACGAGAGGCAGTG |
| AT2G20450 | 60S RIBOSOMAL PROTEIN L14-1 (RPL14A) | UNIQUE for mannitol | TGCAGAAGAGAAGAGCTGCC | TTAGCAAGCTCCTGCCTCAC |
| AT1G20630 | CATALASE 1 (CAT1) | UNIQUE for mannitol | AAGGTTGGATCCAGTTCGCC | CTCCCCTGGTTGCTTGAAGT |
| AT1G77060 | Phosphoenolpyruvate carboxylase family | UNIQUE for mannitol | AACAGACGTGCGTGCTACTT | TCATCGTCGTCTCTTGGTGC |
| AT1G12250 | THYLAKOID LUMENAL PROTEIN TL20.3 (TL20.3) | UNIQUE for mannitol | CGTCTACAGCTAAGTTCCCGT | CGCTGGAACAACAACCTAGT |
| AT1G51680 | 4-COUMARATE:COA LIGASE 1 (4CL1) | UNIQUE for control | CTGATGTTGCTGTTGTCGCA | CTTTTTACCTGTTTCGACACGAAT |
| AT3G02540 | RADIATION SENSITIVE23C (RAD23C) | UNIQUE for control | ATGAACCCGTTGAAGGAGGG | CCTCCCAACGTTTTGCTGTT |
| AT5G35620 | EUKARYOTIC TRANSLATION INITIATION FACTOR ISOFORM 4E (EIF(ISO)4E) | Upregulated | CTCTTTCGAAACCACCACTTGT | AGAGAAGCATTAGGGTGTTGCT |
| AT3G22060 | CYSTEINE-RICH REPEAT SECRETORY PROTEIN 38 (CRRSP38) | Downregulated | CATTCAACTCGCAGACGAAAG | ACCCACAACTCTCCCTCCTTC |
| AT4G30190 | ATPase 2 (AHA2) | Upregulated | CCTCGCATCCATTCCTGTTG | CACCACCTTTGCAATGAACA |

**Supplementary Table S2.** List of primers used for expression analysis after 4 h of mannitol treatment. In addition to the AT-code, gene name, forward and reverse primer, the trend of the protein abundance or phosphorylation event is listed.

| AT-Code | Gene name | Trend | Forward primer | Reverse primer |
|---|---|---|---|---|
| AT3G08770 | NON-SPECIFIC LIPID-TRANSFER PROTEIN (LTP6) | UNIQUE for mannitol | TCTGCTATTGGAGGACTCACTCTC | TGCTTGTCTCACTGGATACTGTCG |
| AT3G54170 | FKBP12-INTERACTING PROTEIN OF 37 kDa (FIP37) | UNIQUE for control | TGAAGTCAGCTGTTCGCGATTTG | AACAACCTCCTCGCCTGCATTG |
| AT3G52920 | Transcriptional activator | UNIQUE for control | ACGTTCAACGAGAAGAATCGTGAG | CTCGCTTTCTCCAACCAACTCC |
| AT1G31860 | HISTIDINE BIOSYNTHESIS 2 (HIS2) | UNIQUE for control | ATGGACTCGACGGTTGTTGACG | CAGCTTCTTCCCTGATCTTTGAGC |
| AT3G20390 | REACTIVE INTERMEDIATE DEAMINASE A (RIDA) | Upregulated | TCATGTTGGCTGATTTGGCTGAC | AGAAGGAGCTGGGAAGTATTTGGC |
| AT1G11870 | SERYL-TRNA SYNTHETASE (SRS) | Upregulated | ATTCCTCTGCAAGGCCTCTGTG | GCACAGCTTCCAAATTAGCATTCG |
| AT5G67030 | ABA DEFICIENT 1 (ABA1) | Upregulated | GGCGACGATTGTTGCGTTTC | CGAGCATGCATCTTCGAAACCTG |
| AT5G65220 | 50S RIBOSOMAL PROTEIN L29 (RPL29) | Downregulated | CTTCGTCTCCAGAAATCGGCAAG | CCGAGCAACTTGTTTCTTCATACG |
| AT3G58500 | PROTEIN PHOSPHATASE 2A ISOFORM 4 (PP2A4) | Downregulated | TTTCCCACTGACAGCCTTGGTG | TCAAGGGTCTCGATGGATGGTG |
| AT3G16640 | TRANSLATIONALLY CONTROLLED TUMOR PROTEIN (TCTP1) | Downregulated | TGAGCTTCTGTCTGACTCTTTCCC | AGCTCCCACAGTAACCCACTTTC |

**Supplementary Table S3.** List of primers used for the genotyping of *aha2-4* and *crrsp38-1* mutant alleles.

| AT-Code | SALK line | Mutant allele name | Left primer | Right primer | Left border primer for T-DNA insertion |
|---|---|---|---|---|---|
| AT4G30190 | SALK_082786 | *aha2-4* | TTGAAAAGGCTGATGGATTTG | CTCCAGGACGTTCAACAAAAG | ATTTTGCCGATTTCGGAAC |
| AT3G22060 | SALK_151902 | *crrsp38-1* | TTTACCGTCGCAACAGTTAGG | TTAAAACGCATCGTTTGGTTC | ATTTTGCCGATTTCGGAAC |

## Statistics

**Supplementary Table S4.** Output of ANOVA test for *crrsp38-1*.

| | Df | Sum Sq | Mean Sq | F value | Pr(>F) |
|---|---|---|---|---|---|
| Treatment | 1 | 1262685 | 1262685 | 704.675 | < 2e-16 |
| Line | 1 | 158693 | 158693 | 88.563 | < 2e-16 |
| Repeat | 3 | 20735 | 6912 | 3.857 | 0.00967 |
| Line:Treatment | 1 | 2049 | 2049 | 1.143 | 0.28563 |
| Residuals | 391 | 700621 | 1792 | | |

**Supplementary Table S5.** Output of Tukey's post hoc test for *crrsp38-1*.

| Interaction | p adj |
|---|---|
| *crrsp38-1*:Control (MS)-Col-0:Control (MS) | 0.0e+00 |
| Col-0:25 mM mannitol-Col-0:Control (MS) | 0.0e+00 |
| *crrsp38-1*:25 mM mannitol-Col-0:Control (MS) | 0.0e+00 |
| Col-0:25 mM mannitol-*crrsp38-1*:Control (MS) | 0.0e+00 |
| *crrsp38-1*:25 mM mannitol-*crrsp38-1*:Control (MS) | 0.0e+00 |
| *crrsp38-1*:25 mM mannitol-Col-0:25 mM mannitol | 2.00E-07 |

**Supplementary Table S6.** Output of ANOVA test for *aha2-4* (16 days after stratification).

| | Df | Sum Sq | Mean Sq | F value | Pr(>F) |
|---|---|---|---|---|---|
| Line | 1 | 14216 | 14216 | 138.175 | <2e-16 |
| Treatment | 1 | 11829 | 11829 | 114.976 | <2e-16 |
| Repeat | 1 | 126 | 126 | 1.226 | 0.27 |
| Line:Treatment | 1 | 3 | 3 | 0.026 | 0.871 |
| Residuals | 169 | 17388 | 103 | | |

**Supplementary Table S7.** Output of Tukey's post hoc test for *aha2-4* (16 days after stratification).

| Interaction | p adj |
|---|---|
| *aha2-4*:Control (MS)-Col-0:Control (MS) | 0.0e+00 |
| Col-0:25 mM mannitol-Col-0:Control (MS) | 0.0e+00 |
| *aha2-4*:25 mM mannitol-Col-0:Control (MS) | 0.0e+00 |
| Col-0:25 mM mannitol-*aha2-4*:Control (MS) | 0.9907543 |
| *aha2-4*:25 mM mannitol-*aha2-4*:Control (MS) | 0.0e+00 |
| *aha2-4*:25 mM mannitol-Col-0:25 mM mannitol | 0.0e+00 |

**Supplementary Table S8.** Output of ANOVA test for *aha2-4* (22 days after stratification).

| | Df | Sum Sq | Mean Sq | F value | Pr(>F) |
|---|---|---|---|---|---|
| Line | 1 | 33847 | 33847 | 19.8 | 1.17E-05 |
| Treatment | 1 | 504802 | 504802 | 295.25 | < 2e-16 |
| Repeat | 3 | 67507 | 22502 | 13.16 | 3.79E-08 |
| Line:Treatment | 1 | 0 | 0 | 0 | 0.99 |
| Residuals | 338 | 577891 | 1710 | | |

**Supplementary Table S9.** Output of Tukey's post hoc test for *aha2-4* (22 days after stratification).

| Interaction | p adj |
|---|---|
| *aha2-4*:Control (MS)-Col-0:Control (MS) | 0.0079799 |
| Col-0:25 mM mannitol-Col-0:Control (MS) | 0.0e+00 |
| *aha2-4*:25 mM mannitol-Col-0:Control (MS) | 0.0e+00 |
| Col-0:25 mM mannitol-*aha2-4*:Control (MS) | 0.0e+00 |
| *aha2-4*:25 mM mannitol-*aha2-4*:Control (MS) | 0.0e+00 |
| *aha2-4*:25 mM mannitol-Col-0:25 mM mannitol | 0.0137891 |

## REFERENCES

**Vu LD, Stes E, Van Bel M, Nelissen H, Maddelein D, Inzé D, Coppens F, Martens L, Gevaert K, De Smet I**. 2016. Up-to-Date Workflow for Plant (Phospho)proteomics Identifies Differential Drought-Responsive Phosphorylation Events in Maize Leaves. Journal of Proteome Research **15**, 4304–4317.
